# Nitric oxide-mediated posttranslational modifications control neurotransmitter release by modulating complexin farnesylation and enhancing its clamping ability

**DOI:** 10.1371/journal.pbio.2003611

**Published:** 2018-04-09

**Authors:** Susan W. Robinson, Julie-Myrtille Bourgognon, Jereme G. Spiers, Carlo Breda, Susanna Campesan, Adrian Butcher, Giovanna R. Mallucci, David Dinsdale, Nobuhiro Morone, Raj Mistry, Tim M. Smith, Maria Guerra-Martin, R. A. John Challiss, Flaviano Giorgini, Joern R. Steinert

**Affiliations:** 1 MRC Toxicology Unit, University of Leicester, Leicester, United Kingdom; 2 Department of Genetics and Genome Biology, University of Leicester, Leicester, United Kingdom; 3 Department of Clinical Neurosciences, University of Cambridge, Cambridge, United Kingdom; 4 Department of Molecular and Cell Biology, University of Leicester, Leicester, United Kingdom; Columbia University, United States of America

## Abstract

Nitric oxide (NO) regulates neuronal function and thus is critical for tuning neuronal communication. Mechanisms by which NO modulates protein function and interaction include posttranslational modifications (PTMs) such as S-nitrosylation. Importantly, cross signaling between S-nitrosylation and prenylation can have major regulatory potential. However, the exact protein targets and resulting changes in function remain elusive. Here, we interrogated the role of NO-dependent PTMs and farnesylation in synaptic transmission. We found that NO compromises synaptic function at the *Drosophila* neuromuscular junction (NMJ) in a cGMP-independent manner. NO suppressed release and reduced the size of available vesicle pools, which was reversed by glutathione (GSH) and occluded by genetic up-regulation of GSH-generating and de-nitrosylating glutamate-cysteine-ligase and S-nitroso-glutathione reductase activities. Enhanced nitrergic activity led to S-nitrosylation of the fusion-clamp protein complexin (cpx) and altered its membrane association and interactions with active zone (AZ) and soluble *N*-ethyl-maleimide-sensitive fusion protein Attachment Protein Receptor (SNARE) proteins. Furthermore, genetic and pharmacological suppression of farnesylation and a nitrosylation mimetic mutant of cpx induced identical physiological and localization phenotypes as caused by NO. Together, our data provide evidence for a novel physiological nitrergic molecular switch involving S-nitrosylation, which reversibly suppresses farnesylation and thereby enhances the net-clamping function of cpx. These data illustrate a new mechanistic signaling pathway by which regulation of farnesylation can fine-tune synaptic release.

## Author summary

One way neurons communicate with each other and with other tissues, such as muscle, is by releasing chemical compounds known as neurotransmitters at sites of interaction known as synapses. This synaptic transmission can be finely regulated by both the releasing neuron and the receiving neuron or muscle cell. Many signaling molecules and pathways are involved in neurotransmitter release. In this study, we have investigated one of such pathways and its role in modulating neurotransmitter release at the neuromuscular synapse of the larva of the fruit fly *Drosophila melanogaster*. This regulation involves nitric oxide, a freely diffusible reactive molecule that can be generated in response to activity in the motor neuron. Several neuronal proteins can be modified by nitric oxide, and our study identified a specific target molecule that regulates neurotransmitter release. This protein, called complexin, undergoes a posttranslational modification in response to increased levels of nitric oxide, changing its localization and function at the synapse and modulating neurotransmission. Our findings can explain how neurons may modulate communication in an activity-dependent manner utilizing nitric oxide signaling.

## Introduction

Throughout the central nervous system (CNS), the volume transmitter nitric oxide (NO) has been implicated in controlling synaptic function by multiple mechanisms, including modulation of transmitter release, plasticity, or neuronal excitability [1–3]. NO-mediated posttranslational modifications (PTMs) in particular have become increasingly recognized as regulators of specific target proteins [4]. S-nitrosylation is a nonenzymatic and reversible PTM resulting in the addition of a NO group to a cysteine (Cys) thiol/sulfhydryl group, leading to the generation of S-nitrosothiols (SNOs). In spite of the large number of SNO-proteins thus far identified, the functional outcomes and mechanisms of the underlying specificity of S-nitrosylation in terms of target proteins and Cys residues within these proteins are not clear.

Synaptic transmitter release is controlled by multiple signaling proteins and involves a cascade of signaling steps [5]. This process requires the assembly of the soluble *N*-ethyl-maleimide-sensitive fusion protein Attachment Protein Receptor (SNARE) complex and associated proteins, the majority of which can be regulated to modulate synapse function. Regulatory mechanisms include phosphorylation of SNARE proteins [6] as well as SNARE-binding proteins such as complexin (cpx), which have been reported at different synapses such as the *Drosophila* neuromuscular junction (NMJ) [7] or in the rat CNS [8].

Several contrasting effects on transmitter release are induced by NO-mediated PTMs [9]. Other forms of protein modification to modulate cellular signaling include prenylation, an attachment of a farnesyl or geranyl-geranyl moiety to a Cys residue in proteins harboring a C-terminal CAAX prenylation motif. This process renders proteins attached to endomembrane/endoplasmic reticulum (ER) and Golgi structures until further processing, as shown for Rab GTPases [10–12]. Farnesylation also regulates mouse cpx 3/4 [13] and *Drosophila* cpx function [14–17]. The Cys within CAAX motifs can also undergo S-nitrosylation, which interferes with the farnesylation signaling [18]; however, direct evidence in a physiological environment is lacking. Cpx function has been studied in many different systems and there is controversy regarding its fusion-clamp activity. Cpx supports Ca^2+^-triggered exocytosis but also exhibits a clamping function [19–24]. Analysis of mouse cpx double-knockout neurons lacking cpx 1 and 2 found only a facilitating function for cpx on release, and different *D*. *melanogaster* and *Caenorhabditis elegans* cpx mutant lines exhibit altered phenotypes in clamping or priming/fusion function [14–17, 24–27], illustrating the controversial actions of cpx.

Here, we investigated the effects of NO on synaptic transmission and found that NO reduces Ca^2+^-triggered release as well as the size of the functional vesicle pool, which was reversed by glutathione (GSH) signaling. At the same time, spontaneous release rates were negatively affected by NO. We confirmed that cpx is S-nitrosylated and that NO changes the synaptic localization of cpx, as also seen following genetic and pharmacological inhibition of farnesylation. Thus, we propose that the function of cpx is regulated by S-nitrosylation of Cys within the CAAX motif to prevent farnesylation. This increases cpx-SNARE-protein interactions, thereby rendering cpx with a dominant clamping function, which suppresses both spontaneous and evoked release.

## Results

### NO-induced suppression of evoked and spontaneous synaptic release is independent of cGMP

**Fig 1:**
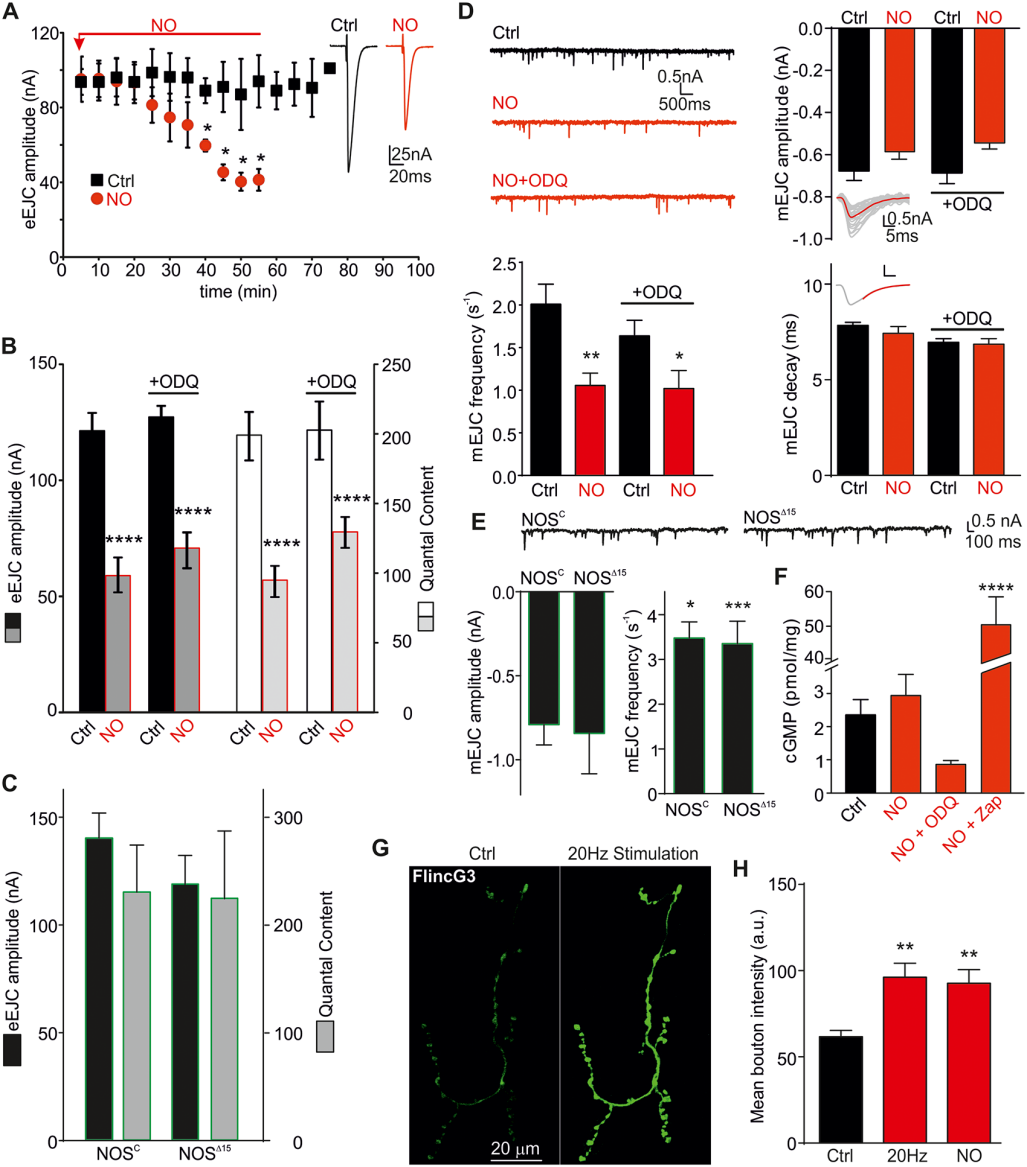
NO reduces evoked release and frequency of spontaneous release in a cGMP-independent manner. (A) NO suppresses evoked release (eEJC) over a time course of 55 min. Insets show representative single eEJCs at 40 min for both conditions. (B) Mean eEJC amplitudes (left axis) and QC (right axis) of *w*^*1118*^ NMJs are reduced following NO exposure (at 40 min). The sGC inhibitor ODQ (50 μM) did not affect the response to NO. (C) Mean eEJC amplitudes (left axis, black) and QC (right axis, grey) of NOS^C^ and NOS^Δ15^ NMJs. (D) Raw mEJC recordings of *w*^*1118*^ NMJs and mEJC parameters (top to bottom: amplitude, frequency, decay). Top insets show representative mEJC recordings. Bottom insets show single mEJCs (grey) and averaged mEJC (red) with single exponential fit to the decay. (E) Raw mEJC recordings for both NOS^C^ and NOS^Δ15^ genotypes. Below, mEJC quantal parameters: amplitude and frequency, Student *t* test each relative to *w*^*1118*^ Ctrl, *p = 0.04, ***p = 0.001. (F) cGMP content of larval brains under the conditions indicated (NO: 40 min NO exposure, NO + ODQ: 40 min NO exposure in the presence of 50 μM ODQ, NO + Zap: 40 min NO exposure + PDE inhibitor Zap, 20 μM). (G) FlincG3 fluorescence images of a Ctrl and stimulated NMJ (20 Hz for 10 s, duty cycle: 1 min for total of 10 min). (H) Summary of FlincG3 fluorescence (in a.u.’s). The raw data can be found in S1 Data. Data denote mean ± SEM in all graphs, ANOVA with post hoc Tukey-Kramer, *p < 0.05, **p < 0.01, ****p < 0.0001. a.u., arbitrary unit; cGMP, cyclic guanosine monophosphate; Ctrl, control; eEJC, evoked EJC; EJC, excitatory junction current; mEJC, miniature EJC; NMJ, neuromuscular junction; NO, nitric oxide; ODQ, 1H-[1,2,4]oxadiazolo[4,3-a]quinoxalin-1-one; PDE, phosphodiesterase; QC, quantal content; sGC, soluble guanylyl cyclase; Zap, zaprinast.

Previously, we found that enhancing endogenous nitric oxide synthase (NOS) activity induced by overexpression of *D*. *melanogaster* NOS (*Dm*NOS) caused a reduction in synaptic strength at the *Drosophila* NMJ synapse [28]. To examine the effects of NO on glutamatergic transmission in more detail, we exposed wild-type (WT) *w*^*1118*^ control (Ctrl) larvae to NO donors, which provide an estimated NO concentration of about 200 nM [29]. When recording evoked excitatory junction currents (eEJCs) up to 70 min during NO incubation, the amplitudes started to decline significantly after 35 min (Fig 1A and S1 Data, *p* < 0.05; *n* = 3 each). Mean eEJC amplitudes and quantal content (QC) at 50 min for Ctrl (122 ± 7 nA, QC: 200 ± 15, *n* = 20–22) and NO treatment (59 ± 7 nA, QC: 93 ± 10, *n* = 14) are shown in Fig 1B. As the canonical NO-cGMP pathway is active in *Drosophila* [30] and potentially responsible for this observation, we blocked the soluble guanylyl cyclase (sGC) with 1H-[1,2,4]oxadiazolo[4,3-a]quinoxalin-1-one (ODQ, 50 μM). Interestingly, ODQ did not prevent the effects of NO, suggesting a cGMP-independent mechanism (amplitudes: Ctrl + ODQ: 127 ± 5 nA, NO + ODQ: 70 ± 7 nA, QC: Ctrl + ODQ: 200 ± 22, NO + ODQ: 130 ± 11, Fig 1B, *n* = 10–16). As *Drosophila* has endogenous NO signaling and produces neuronal NO in a Ca^2+^/calmodulin-dependent manner [31, 32], we used NOS knockout-like (NOS “null”) larvae to assess endogenous NO modulation of release. We used two different lines with strongly reduced *Dm*NOS showing NOS “null” activity (NOS^C^ and NOS^Δ15^ [33, 34]) and we would expect that lack of endogenous NO generation has the opposite effects on release. When recording eEJCs, both genotypes exhibited a tendency towards larger eEJC amplitudes and QC (Fig 1C) and, in addition, we detected an increased presynaptic release probability (*p*_*vr*_) in NOS^C^ NMJs, as indicated by the reduced paired pulse ratio (PPR) at 20 ms ISI (0.80 ± 0.03 [*n* = 11], *p* = 0.002, Student *t* test) compared to WT Ctrls (0.93 ± 0.03 [*n* = 17]), indicating endogenous nitrergic effects on release probabilities.

To further understand the effects of NO on release, we analyzed miniature EJCs (mEJCs) under the same conditions. NO had no effect on mEJC amplitudes or decay kinetics; however, the frequency was reduced following NO and NO+ODQ incubation (Ctrl: 2.0 ± 0.2 nA [*n* = 25], NO: 1.1 ± 0.1 nA [*n* = 16], NO+ODQ: 1.0 ± 0.2 nA [*n* = 8], ODQ: 1.7 ± 0.2 nA [*n* = 11], Ctrl versus NO: *p* < 0.01, Ctrl versus NO+ODQ: *p* < 0.05, Fig 1D). This suggests that NO is unlikely to affect synaptic vesicle filling or composition/activity and density of postsynaptic *D*. *melanogaster* glutamate receptors (*Dm*GluR) [35]. We tested miniature events in the NOS “null” mutants and confirmed a further inhibitory role of NO signaling on release, with mEJC frequencies being significantly enhanced in NOS^Δ15^ (3.5 ± 0.5 s^−1^ [*n* = 4], *p* = 0.001) and NOS^C^ (3.5 ± 0.4 s^−1^ [*n* = 16], *p* = 0.04) larvae compared to Ctrl (Fig 1E), without affecting mEJC amplitudes (NOS^Δ15^: 0.8 ± 0.1 nA [*n* = 13], NOS^C^: 1.1 ± 0.3 nA [*n* = 3] Fig 1E) or decay kinetics (NOS^Δ15^: 8.9 ± 0.6 ms [*n* = 12], NOS^C^: 9.4 ± 0.3 ms [*n* = 4], *p* > 0.05 versus Ctrl). Thus, reduction of endogenous NOS activity shows opposite effects to elevation of NO levels, confirming the inhibitory action of NO on evoked and spontaneous vesicle release.

As the data imply cGMP-independent signaling, we wanted to confirm that cGMP levels are not altered following NO stimulation. Thus, we measured cGMP directly in isolated larval brains. NO application did not raise cGMP levels (at 50 min: Ctrl: 2.4 ± 0.5 pmol/mg, NO: 3.0 ± 0.6 pmol/mg, *p* > 0.05 [*n* = 30 each], Fig 1F). Cyclase inhibition in the presence of NO did not significantly reduce cGMP levels, confirming lack of NO-induced neuronal cGMP accumulation. We found that any generated cGMP was broken down by phosphodiesterase *Dm*PDE5/6 [36], as cGMP increased following NO stimulation only with PDE inhibition (20 μM zaprinast [Zap]; NO+Zap: 50.2 ± 8.3 pmol/mg, *p* < 0.0001), while Zap alone had no effect (Zap: 4.6 ± 2.0 pmol/mg, *p* > 0.05).

To assess whether NO is produced endogenously to induce modulation of synaptic function as observed above, we expressed FlincG3 presynaptically and stimulated NMJs at 20 Hz (for 10 s every minute for 20 min). As shown in Fig 1G, 20 Hz stimulation induced a significant increase in fluorescence, confirming endogenous presynaptic generation of NO (Ctrl: 62 ± 4 arbitrary units [a.u.’s], Stim: 96 ± 8 a.u.’s, Fig 1H [*n* = 13–15 boutons], *p* < 0.01). Importantly, addition of the NO donor did not further increase the fluorescence, indicating that activity-induced synaptic NO concentrations reach similar levels (NO: 93 ± 7 a.u.’s). A potential target of NO signaling is mitochondria [37], which are required for the energy to maintain vesicle recycling and synaptic transmission [38]. Thus, we measured mitochondrial activity in third instar larvae under the same conditions (50 min NO incubation) and found that mitochondrial activity was unaffected by NO (S1 Fig and S9 Data), suggesting that the effects of NO on synaptic transmission are not due to ATP depletion. Together, these data suggest that NO has a presynaptic effect on transmitter release, which is independent of cGMP signaling.

### Ca^2+^ dependency of evoked release is reduced by NO

Several mechanisms contribute to the regulation of synaptic strength [39], including altered *p*_*vr*_, alterations in the number of readily releasable vesicles and release sites (*N*) or quantal size (*q*). Alterations in *q* are likely not involved in the NO-induced effects observed based on our mEJC data above (Fig 1). We next assessed additional release parameters, including *p*_*vr*_, *N*, vesicle pool size, and Ca^2+^ dependency of release in NOS “null” and WT NMJs following nitrergic signaling.

**Fig 2:**
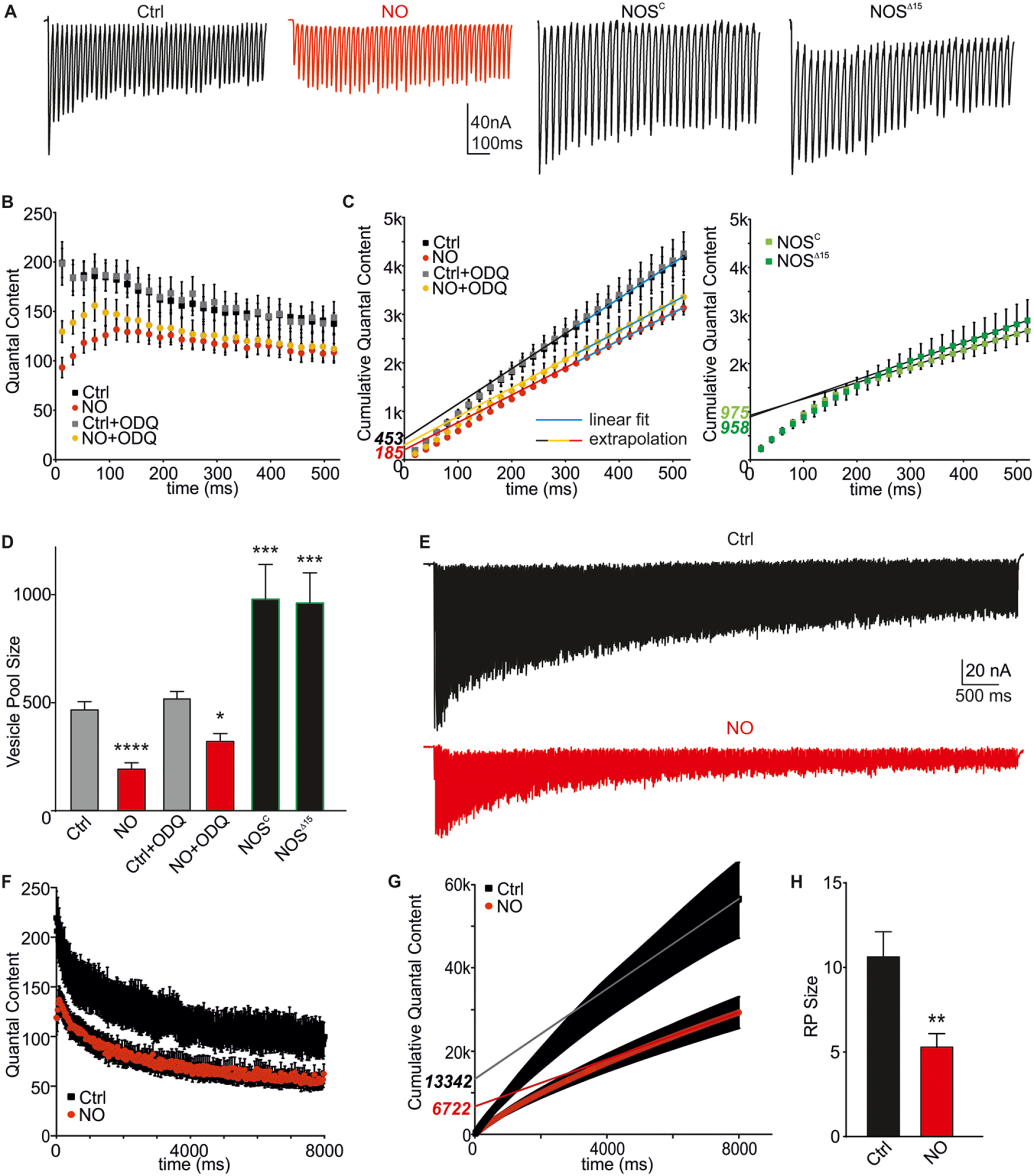
The size of the RRP and RP is reduced by NO and enhanced in NOS “null” NMJs. (A) Raw eEJC recordings of a 50-Hz train, 500 ms. (B) Mean QC for conditions indicated. (C) Cumulative QC with back extrapolated linear regression to the last 200 ms to time 0, yielding an estimated readily releasable vesicle pool size (RRP, intercept with y-axis, pool sizes in italics). (D) Mean RRP sizes for conditions indicated. NO reduces PPR sizes, with ODQ having no effects. NOS “null” NMJs show potentiated responses (ANOVA with post hoc Tukey-Kramer was used for comparisons). (E) Raw eEJC recordings of a 50-Hz train, 8 s. (F) Mean QC for conditions indicated. (G) Cumulative QC with back extrapolated linear regression to the last 2 s to time 0 (*w*^*1118*^ Ctrl and NO), yielding an estimated RP size (intercept with y-axis, pool sizes in italics). (H) Mean RP sizes for conditions indicated. NO reduces RP sizes similarly to RRP sizes. The raw data can be found in S2 Data. Student *t* test, **p = 0.0062, data denote mean ± SEM in all graphs. Ctrl, control; eEJC, evoked EJC; EJC, excitatory junctional current; NMJ, neuromuscular junction; NO, nitric oxide; NOS, nitric oxide synthase; ODQ, 1H-[1,2,4]oxadiazolo[4,3-a]quinoxalin-1-one; PPR, paired pulse ratio; QC, quantal content; RP, reserve pool; RRP, readily releasable pool.

We determined pool size via a method successfully applied at the *Drosophila* NMJ, by analyzing the cumulative QC of trains of higher frequency stimulation [40]. Stimulation at 50 Hz for 500 ms in 1.5 mM extracellular calcium concentration ([Ca^2+^]_e_) retrieves vesicles from the readily releasable pool (RRP) [41]. This stimulation pattern induced mild depression in Ctrls and strong initial facilitation of trains under NO conditions (Fig 2A and S2 Data). Cumulative QC analysis revealed a pool size of 453 ± 37 (*n* = 17) in Ctrl and 185 ± 18 in NO-exposed NMJs (*n* = 16, *p* < 0.01), suggesting a strong reduction in ready-releasable/recycling vesicles (Fig 2A–2D). Supporting the above data, pool size estimation in the presence of ODQ confirmed cGMP independence (NO+ODQ: 310 ± 33 [*n* = 9], *p* < 0.05 versus Ctrl; ODQ alone: 501 ± 34 [*n* = 9], *p* > 0.05 versus Ctrl, Fig 2A–2D). And importantly, analysis of the vesicle pool sizes in NOS “null” mutants revealed a strong 2-fold increase compared to *w*^*1118*^ Ctrl and an over 5-fold increase compared to NO application (NOS^C^: 975 ± 161 [*n* = 11]; NOS^Δ15^: 958 ± 139 [*n* = 4], *p* < 0.001 versus Ctrl, Fig 2A–2D). To exclude any potential developmental effects caused by NOS deficiency that could account for these strong increases in release, we assessed NMJ morphology and ultrastructure. We analyzed the total volume of NMJs (horseradish peroxidase [HRP] signal) and the number of Bruchpilot (Brp) puncta/NMJ volume of z-stack confocal images (S2A and S2B Fig and S9 Data) and measured the number of AZs, T-bars per Ib bouton, and vesicles within a 250-nm semicircle around the AZ (S2C and S2D Fig). These data indicated that reduced NOS activity has no developmental impact on the structure of NMJs and synaptic boutons and can therefore not explain the physiological differences observed above.

In addition to changes in release, NO could also exert its effects indirectly via modulating transmitter uptake and pool recovery. To exclude this possibility that altered recovery from depression affected the above pool estimations, we examined eEJC recovery. Following depletion of vesicle pools during a 50-Hz train (1 s), we measured the time course of recovery over the following 60 s. NO did not show any effects on the time constant of recovery (S3 Fig and S9 Data).

In order to test whether NO acts specifically on RRP or also affects the availability of other pools, we stimulated the NMJ for longer periods (8 s) at 50 Hz. This prolonged stimulation leads to recruitment of vesicles from the reserve pool (RP) [42, 43]. Analysis revealed that NO also caused a strong reduction of release from the RP (Fig 2E–2H, Ctrl: 11,160 ± 1,645 [*n* = 6]; NO: 5,286 ± 798 [*n* = 7], *p* = 0.0062).

One important protein that regulates vesicle clustering and release of neurotransmitter is the phosphoprotein synapsin (syn), which regulates recycling of RP vesicles in *Drosophila* NMJs [43]. We tested whether modulation of syn could be responsible by employing larvae deficient in this protein from the *Syn*^*97*^-null mutation [44]. These larvae did not exhibit any reduction in single-stimulus QC compared to Ctrls, but prolonged recruitment (500 ms at 50 Hz) showed reduced vesicle availabilities. Importantly, incubation of *Syn*^*97*^ larvae with NO led to further reduction of both parameters (S4 Fig and S9 Data), suggesting that NO effects are via a different signaling route.

Based on these data, we suggest that NO decreases release of vesicles from the RRP and RP but does not affect the rate of vesicle pool recovery from depletion. The NO-mediated effects appear to be independent of syn, suggesting an event downstream of vesicle recruitment per se.

**Fig 3:**
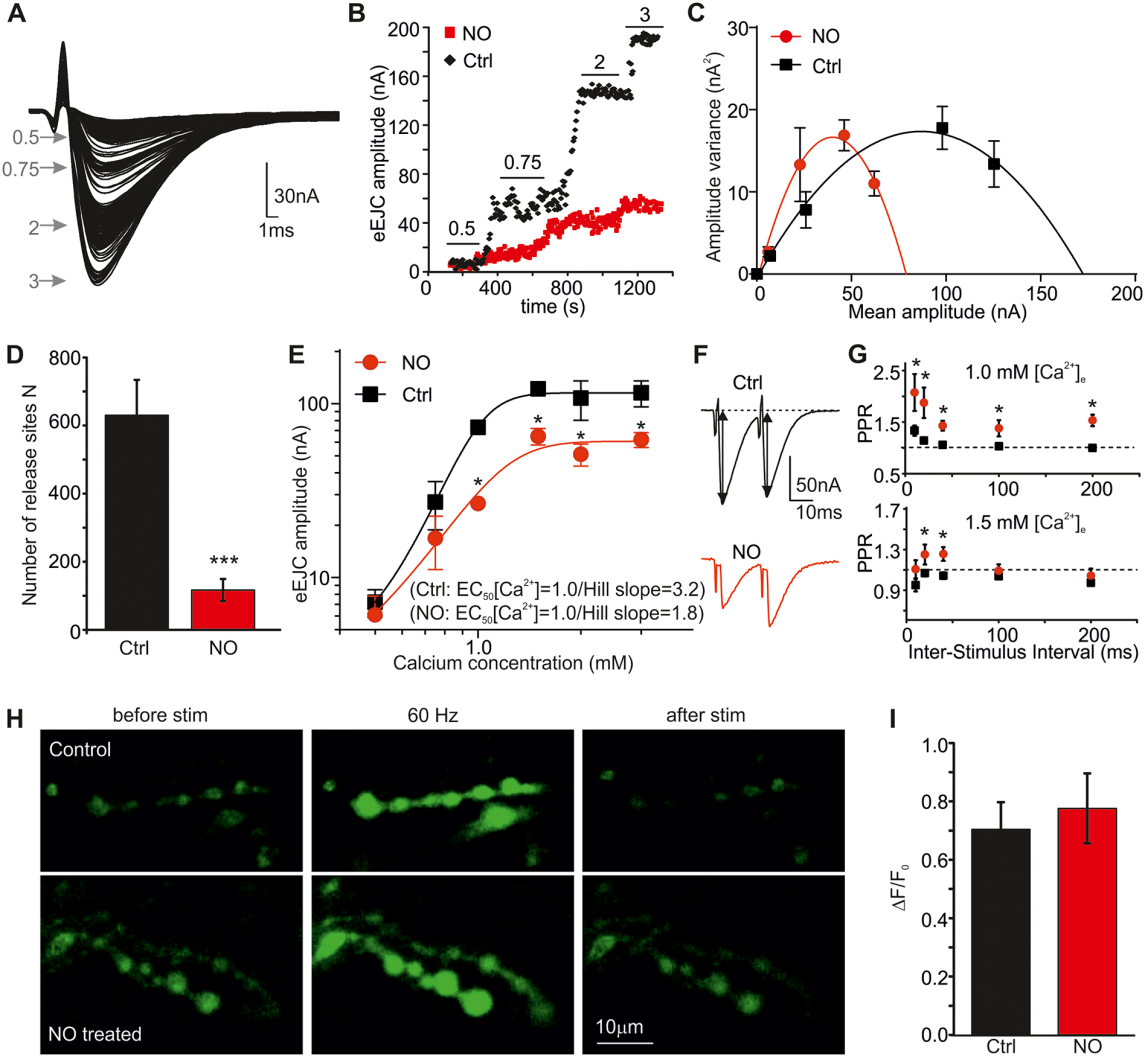
Ca^2+^ dependency of evoked release is reduced by NO. (A) Representative raw eEJC recordings at different [Ca^2+^]_e_ (from 0.5 to 3 mM). (B) Time course of single eEJCs for 2 NMJs (*w*^*1118*^ Ctrl and NO exposure) at indicated [Ca^2+^]_e_. (C) Parabolic fits to the variance-mean relationships for the conditions indicated. (D) *N* estimated from fluctuation analysis in both conditions. (E) Ca^2+^ cooperativity of evoked release is shown on a double logarithmic plot for *w*^*1118*^ Ctrl and NO. (F) Raw traces for PPRs of a Ctrl and NO-treated NMJ at 1.0 and 1.5 mM [Ca^2+^]_e_, illustrating a reduced release probability following NO exposure. (G) Summary of PPR at 1 and 1.5 mM [Ca^2+^]_e_ for various ISI for Ctrl and NO (*n* = 5 larvae for Ctrl, *n* = 3 larvae for NO, with 2–3 NMJs per larva). (H) Images of myrGCaMP-expressing NMJs from a Ctrl (top) and a NO-treated larva (bottom) before, during (at 3 s, 60 Hz), and after the 8-s train. Changes in GCaMP5 fluorescence were analyzed for each bouton to calculate ΔF/F_0_. The raw data can be found in S3 Data. Values per NMJ were averaged and data denoting mean ± SEM for each condition of 6–8 NMJs (3–4 larvae) are shown in I. *p < 0.05, ***p < 0.001, Student *t* test. [Ca^2+^]_e_, extracellular calcium concentration; Ctrl, control; eEJC, evoked EJC; EJC, excitatory junction current; ISI, interspike interval; myrGCaMP, *N*-myristoylated GCaMP; *N*, number of release-ready vesicles; NMJ, neuromuscular junction; NO, nitric oxide; PPR, paired pulse ratio; stim, stimulation.

We next applied an independent approach to estimate the synaptic parameters: fluctuation analysis [45] to estimate the number of functional release sites *N*. eEJCs were elicited at varying calcium concentrations ([Ca^2+^]_e_: 0.5–3 mM, 0.2 Hz) and amplitudes were plotted over [Ca^2+^]_e_ (Fig 3A and 3B and S3 Data). NO exposure led to reduced release across different Ca^2+^ concentrations (0.75–3 mM). *N* was estimated from parabolic fits to the variance-mean plots for each NMJ (Fig 3C). This analysis revealed a strong reduction in *N* following NO exposure (Fig 3D, *N*_Ctrl_: 630 ± 104 [*n* = 5], *N*_NO_: 117 ± 32 [*n* = 6], *p* = 0.0006). The estimation of *N* from the fluctuation analysis (about 600) in Ctrl is in accordance with previously reported electron microscopy (EM) data showing a number of about 500 vesicles per NMJ [46]. These data confirm that NO most likely reduces the number of releasable vesicles by preventing vesicle fusion at individual release sites.

The reduced QC seen following NO exposure can also be attributable to a change in the Ca^2+^ dependency of release, so we determined whether the reduced transmitter release is due to altered Ca^2+^ cooperativity of release [47]. The Hill slope was strongly reduced by NO (Ctrl: 3.2 ± 0.4 [*n* = 6], NO: 1.8 ± 0.7 [*n* = 5], *p* = 0.0024, Fig 3E); however, the half maximal effective Ca^2+^ concentration (EC_50_) was unaltered (Ctrl: 1.0 ± 0.03, NO: 1.0 ± 0.09, *p* > 0.05, Fig 3E), indicating that sensitivity to Ca^2+^ was not affected by NO.

To further assess nitrergic effects on *p*_*vr*_, we used the PPR approach by delivering two pulses with interspike intervals (ISIs) between 10 and 200 ms at two different [Ca^2+^]_e_ (1 and 1.5 mM, Fig 3F and 3G) in Ctrl and NO-treated NMJs. Analysis showed that Ctrl NMJs only exhibit slight potentiation at low Ca^2+^ and high ISI, indicative of low *p*_*vr*_. In contrast, *p*_*vr*_ in the presence of NO was decreased, as shown by an increased PPR (potentiation at all ISI at 1 mM Ca^2+^ and 20 and 40 ms ISI at 1.5 mM Ca^2+^, *p* < 0.05, Ctrl versus NO at each ISI), which is also in agreement with elevated *p*_*vr*_ in NOS “null” larvae. With about 500 release sites per NMJ and a QC of 200 (Ctrl) and 90 (NO), our data present estimated *p*_*vr*_ values of 0.33 (Ctrl) and 0.16 (NO), with Ctrl values similar to estimates made previously in WT larvae [40].

Previously, we have shown that NO signaling can suppress mammalian P/Q and N-type Ca^2+^ channels [48]. In order to test whether altered Ca^2+^ influx could cause the observed effects on evoked release at the NMJ, we tested whether NO application for 60 min changed presynaptic Ca^2+^ levels during a train of synaptic stimulation. GCaMP5 was expressed presynaptically and activity-evoked Ca^2+^ influx in type 1b NMJ boutons was imaged at different extracellular Ca^2+^ concentrations (0.25–3 mM). Our data showed that NO had no effect on stimulated Ca^2+^ levels at any concentration tested (ΔF/F_0_, myrGCamP5: 3 mM Ca^2+^: Ctrl: 0.70 ± 0.09, NO: 0.78 ± 0.12 [*n* = 13–18 boutons from 4–6 NMJs each], *p* > 0.05; Fig 3H and 3I; GCaMP5: 0.25 mM Ca^2+^: 0.24 ± 0.03, NO: 0.14 ± 0.03, 0.5 mM Ca^2+^: Ctrl: 0.42 ± 0.08, NO: 0.50 ± 0.07, 1.5 mM Ca^2+^: Ctrl: 1.13 ± 0.14, NO: 1.18 ± 0.21 [*n* = 28–46 boutons from 7–11 NMJs each], *p* > 0.05; S5 Fig and S9 Data). Together, the data suggest that NO reduced evoked release and the frequency of spontaneous release, likely due to reduced release probability and Ca^2+^ cooperativity, which manifests itself in reduced vesicle fusion. We showed that the Ca^2+^ dependence of release, but not Ca^2+^ entry per se, was reduced by NO, which indicates a possible modulation of SNARE (-associated) protein interactions via NO-mediated PTMs.

### Enhanced denitrosylation signaling reverses and precludes NO effects

S-nitrosylation is a reversible non-enzymatic protein modification, the levels of which can be regulated via S-nitrosoglutathione reductase (GSNOR), the sole alcohol dehydrogenase 5 (ADH-5) isozyme in vertebrate brains [49], which has a homologue in *Drosophila* (encoded by the formaldehyde dehydrogenase [*fdh*] gene). This de-nitrosylation process requires GSH. GSH is produced from L-glutamate and Cys via the enzyme glutamate-cysteine ligase (GCL), the rate-limiting step in GSH synthesis in fly [50]. The *Drosophila* GCL holoenzyme is heterodimeric, consisting of a catalytic (*Dm*GCLc) and a modifier (*Dm*GCLm) subunit, each encoded by a unique gene, and overexpression of either subunit increases cellular GSH levels [50].

**Fig 4:**
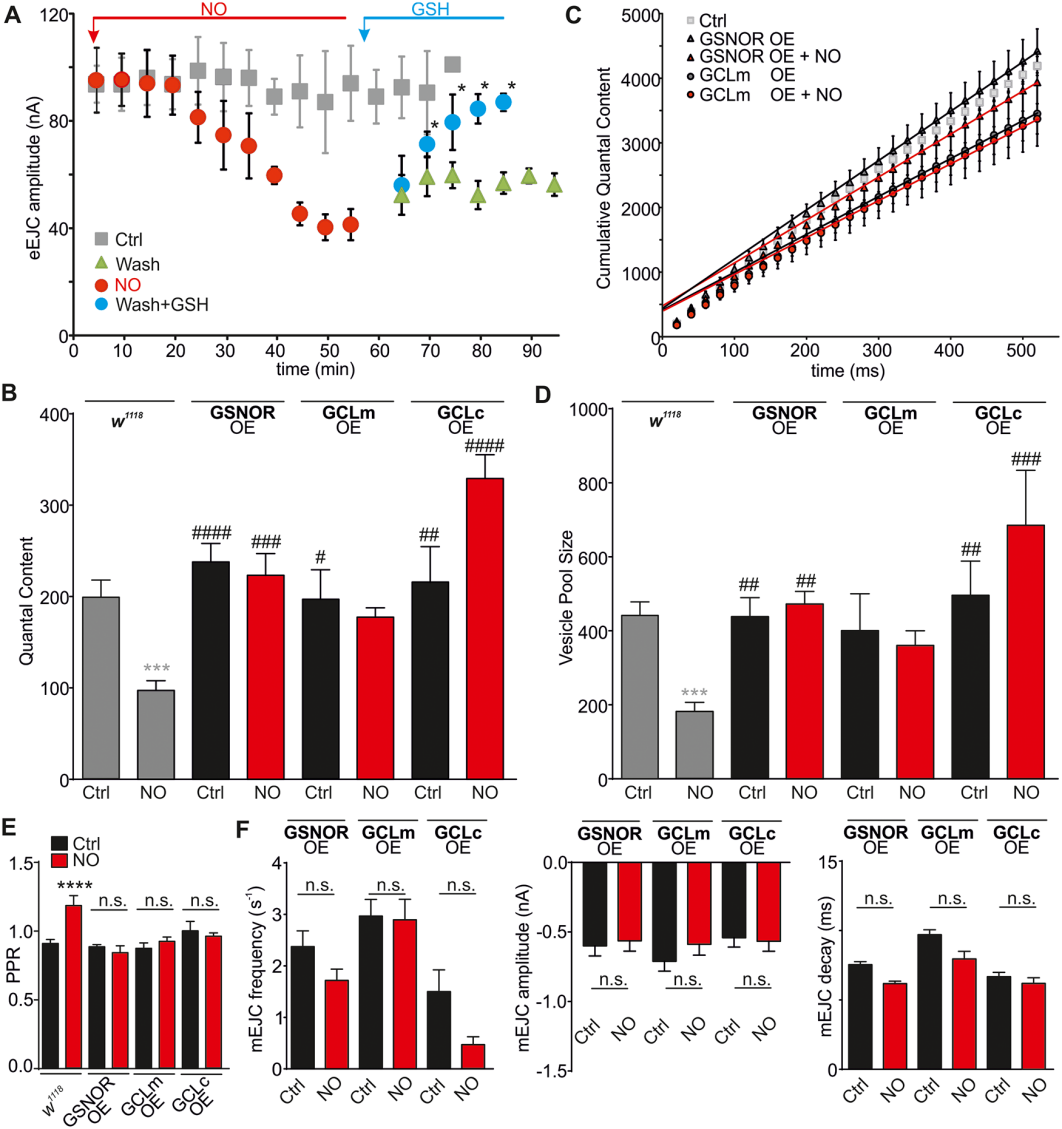
Genetic and pharmacological induction of denitrosylation reverses and prevents nitrergic effects on transmitter release and vesicle pools. (A) NO-induced suppression of evoked release (*w*^*1118*^ Ctrl eEJCs data in grey from Fig 1A) can be reversed after 15 min of GSH application (150 μM, blue). Wash out of NO alone shows mild nonsignificant recovery (green). (B) Mean QC for conditions indicated (OE, *elav* > UAS-*fdh*31 [GSNOR], *elav* > UAS-*GCLm*, *elav* > UAS-*GCLc*, all ± NO). NO has no effects on QC in genotypes indicated (*w*^*1118*^ + NO data in grey from Fig 1 for comparisons, ***p < 0.0001 *w*^*1118*^ + NO versus *w*^*1118*^), #*p* < 0.05, ##*p* < 0.01, ###*p* < 0.001, ####*p* < 0.0001 versus *w*^*1118*^ + NO. (C) Cumulative QC graphs for genotypes indicated (±NO) with linear regression to the last 200 ms, Ctrl in grey taken from Fig 2 for comparison. (D) Mean RRP sizes, NO has no effects on vesicle pool size in tested genotypes (*w*^*1118*^ data in grey from Fig 1, *w*^*1118*^ + NO versus *w*^*1118*^: ***p < 0.0001), ##*p* < 0.01, ###*p* < 0.01, ####*p* < 0.01 versus *w*^*1118*^ + NO. (E) PPR at 20 ms ISI for indicated conditions, ****p < 0.0001 versus Ctrl. (F) mEJCs analysis: left, mean mEJC amplitudes (±NO), middle, mean mEJC frequencies (±NO), right, mean mEJC decay kinetics for genotypes indicated (±NO). OE of GSNOR, GCLm, or GCLc prevents NO effects on the frequency of mEJCs. The raw data can be found in S4 Data. Data denote mean ± SEM for all data comparisons. ANOVA with post hoc Tukey-Kramer. Ctrl, control; eEJC, evoked EJC; EJC, excitatory junction current; *fdh*, formaldehyde dehydrogenase; *GCLc*, glutamate-cysteine ligase catalytic subunit C; *GCLm*, glutamate-cysteine ligase catalytic subunit M; GSH, glutathione; GSNOR, S-nitrosoglutathione reductase; ISI, interspike interval; mEJC, miniature EJC; NO, nitric oxide; n.s., nonsignificant; OE, overexpression; PPR, paired pulse ratio; QC, quantal content; RRP, readily releasable pool.

In order to assess the contributions of SNO formation to the physiology at the NMJ, we investigated the effects of altering neuronal GSH levels. If NO mediates its observed actions via SNO formation, we should be able to prevent/reduce the effects on transmitter release by providing elevated GSH levels by (i) GSH supplementation, (ii) overexpression of GSNOR (*fdh*), or (iii) overexpression of GCL (*Dm*GCLm/c) and, inversely, enhance NO effects by using RNA interference (RNAi) expression of the above proteins. We tested first the recovery of NO-mediated reduction of eEJC amplitudes following NO exposure for 50 min by washing out NO. eEJC amplitudes recovered slightly (Fig 4A, green and S4 Data); however, when washing in GSH (150 μM), the amplitudes recovered to control levels after 15 min (GSH [blue] versus NO at 50 min [red], *p* < 0.05), indicating a GSH-mediated reversal.

To characterize effects of endogenous GSH formation, we used *elav*-Gal4-driven UAS-*fdh*31, UAS-*Dm*GCLm, and UAS-*Dm*GCLc overexpression. It has been shown that overexpression of either *Dm*GCLc or *Dm*GCLm results in enhanced enzyme activity and elevated GSH levels [50], GSNOR overexpression (*elav* > UAS-*fdh*31) reduces global S-nitrosylation in fly, and conversely, GSNOR-RNAi expression (*elav* > UAS-*fdhri*34) elevates SNO protein levels [51]. Overexpression of GSNOR and GCLm/c (Fig 4B–4D) prevented NO effects on QC (GSNOR: 238 ± 20 [*n* = 11], *Dm*GCLm: 197 ± 32 [*n* = 7], *Dm*GCLc: 215 ± 39 [*n* = 6], GSNOR+NO: 223 ± 24 [*n* = 8], *Dm*GCLm+NO: 177 ± 10 [*n* = 7], *Dm*GCLc+NO: 329 ± 26 [*n* = 3], *p* > 0.05) and vesicle pool sizes (GSNOR: 438 ± 51 [*n* = 10], *Dm*GCLm: 400 ± 99 [*n* = 7], *Dm*GCLc: 496 ± 93 [*n* = 6], GSNOR+NO: 472 ± 34 [*n* = 8), *Dm*GCLm+NO: 360 ± 40 [*n* = 7], *Dm*GCLc+NO: 685 ± 148 [*n* = 3], *p* > 0.05, Fig 4B–4D). These data confirm that by enhancing GSNOR and GCL activities, thereby elevating intracellular GSH levels, the effects of NO on pool size and *p*_*vr*_ (PPR at 20 ms ISI; *w*^*1118*^ Ctrl [0.93 ± 0.03] versus NO [1.2 ± 0.07], *p* < 0.0001, GSNOR overexpression [0.88 ± 0.02], +NO [0.84 ± 0.05]/GCLm overexpression [0.87 ± 0.04], +NO [0.92 ± 0.03]/GCLc overexpression [0.99 ± 0.07], +NO [0.96 ± 0.02], *p* > 0.05, Fig 4E) were precluded, suggesting that this was due to reduced SNO formation. Furthermore, overexpression of GSNOR, *Dm*GCLm, and *Dm*GCLc prevented the reduction in mEJC frequency following NO exposure (f_GSNOR_: 2.4 ± 0.3 s^−1^ [*n* = 13]; f_*Dm*GCLm_: 3.0 ± 0.3 s^−1^ [*n* = 13]; f_*Dm*GCLc_: 1.5 ± 0.42 s^−1^ [*n* = 5]; f_GSNOR_+NO: 1.7 ± 0.2 s^−1^ [*n* = 7]; f_*Dm*GCLm_+NO: 2.9 ± 0.4 s^−1^ [*n* = 13]; f_*Dm*GCLc_+NO: 0.4 ± 0.1 s^−1^ [*n* = 3], *p* > 0.05 versus *w*^*1118*^ Ctrl and versus each Ctrl, Fig 4F) without affecting mEJC amplitudes (GSNOR: −0.6 ± 0.07 nA [*n* = 13]; *Dm*GCLm: −0.7 ± 0.07 nA [*n* = 13]; *Dm*GCLc: −0.5 ± 0.07 nA [*n* = 5]; GSNOR+NO: −0.6 ± 0.07 nA [*n* = 7]; *Dm*GCLm+NO: −0.6 ± 0.08 nA [*n* = 13]; *Dm*GCLc+NO: −0.6 ± 0.07 nA [*n* = 3], *p* > 0.05 versus *w*^*1118*^ Ctrl and versus each Ctrl, Fig 4F) or decays (GSNOR: 7.5 ± 0.2 ms [*n* = 13]; *Dm*GCLm: 9.7 ± 0.4 ms [*n* = 13], *Dm*GCLc: 6.7 ± 0.3 ms [*n* = 5], GSNOR+NO: 6.2 ± 0.2 ms [*n* = 7], *Dm*GCLm+NO: 7.9 ± 0.5 ms [*n* = 13], *Dm*GCLc+NO: 6.2 ± 0.4 ms [*n* = 3], *p* > 0.05 versus *w*^*1118*^ Ctrl and versus each Ctrl, Fig 4F). Furthermore, the reduction of endogenous GSNOR and *Dm*GCLm activities (*elav* > UAS-RNAi) caused partial electrophysiological phenotypes, such as a decrease in eEJC amplitudes, QC, or vesicle pool size compared to *w*^*1118*^ Ctrl, with NO having no further major negative effects (S6 Fig and S9 Data).

### Nitrergic effects require the presence of cpx

We next asked which signaling routes and PTMs are involved in NO modulation of release. The SNARE-binding and fusion-clamp protein cpx regulates not only the Ca^2+^ cooperativity of evoked release but also spontaneous release [14] as well as release probabilities [52], thereby presenting a strong candidate for mediating the observed NO-induced changes.

**Fig 5:**
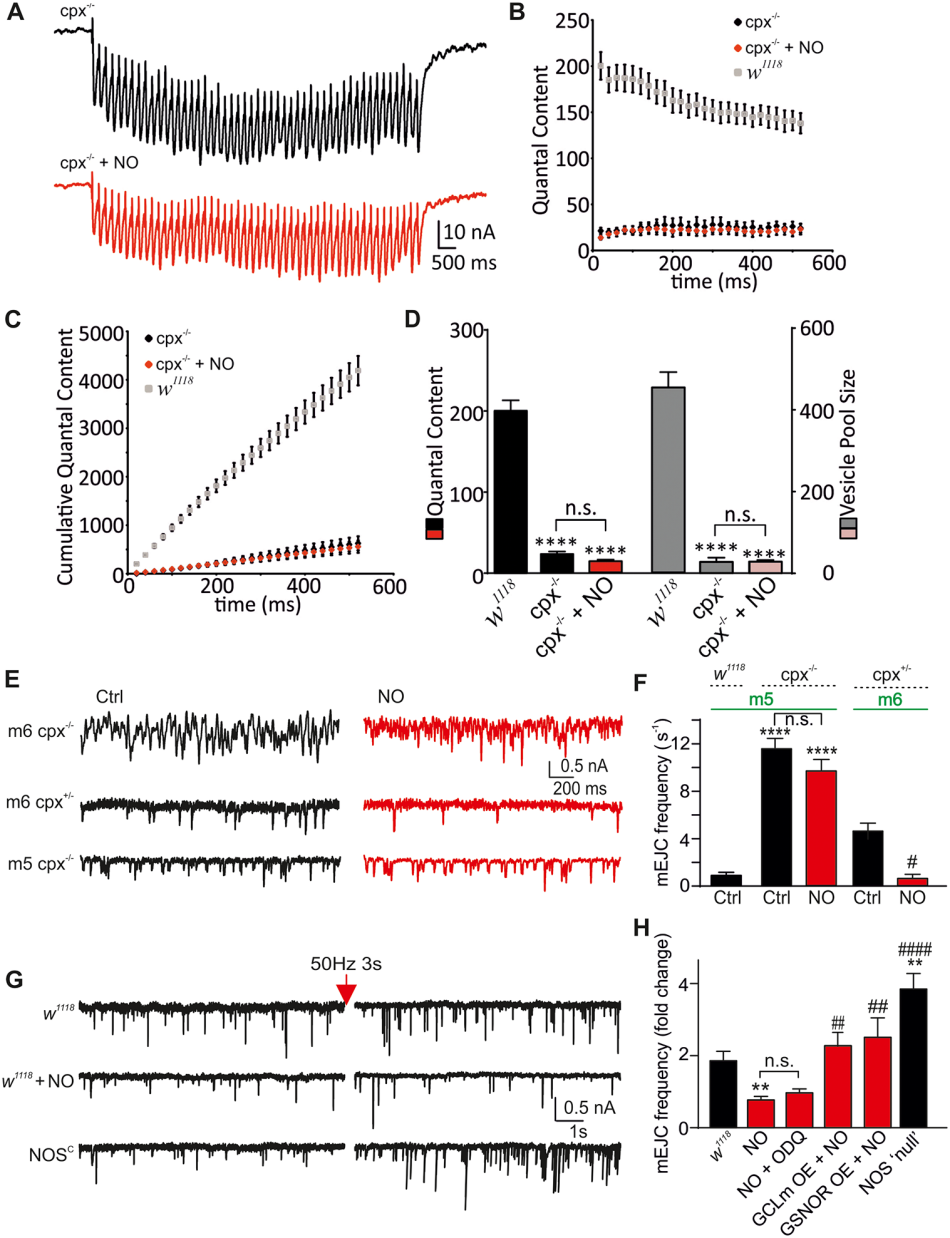
Nitrergic effects on evoked and spontaneous release require cpx. (A) Raw eEJC recordings of a 50-Hz train, 500 ms in cpx^-/-^ larvae (±NO). (B) Mean QC for genotypes indicated, showing the lack of NO effects in cpx^-/-^ larvae (*w*^*1118*^ Ctrl in grey from Fig 2). (C) Cumulative QC for the same conditions as in (B). (D) Mean QC and vesicle pool sizes for the genotypes indicated (*w*^*1118*^ Ctrl data from Figs 1 and 2, ****p < 0.0001 versus *w*^*1118*^ Ctrl). (E) mEJCs recordings from m6 and m5 in cpx^-/-^ and cpx^+/-^ ± NO. (F) Mean mEJC frequencies for conditions and genotypes indicated (****p < 0.0001 versus m5 *w*^*1118*^ Ctrl, #*p* < 0.05 versus m6 cpx^+/-^ Ctrl). (G) Raw traces of mEJC recordings before (left) and after (right) high frequency stimulation in *w*^*1118*^ (±NO) and NOS^C^ larvae. (H) Average fold change of mEJC frequency during 50 s after stimulation compared to baseline frequency before stimulation for conditions and genotypes indicated (NOS “null” comprised of data from NOS^C^ and NOS^Δ15^). NO treatment (40 min) suppresses increases in frequency, NOS “null” potentiates relative increases and enhanced denitrosylation (GCLm and GSNOR OE) prevents NO-induced suppression (*p < 0.05, **p < 0.01 versus Ctrl, ##*p* < 0.01, ####*p* < 0.001 versus NO). The raw data can be found in S5 Data. Data denote mean ± SEM in all graphs, ANOVA with post hoc Tukey-Kramer. Ctrl, control; cpx, complexin; eEJC, evoked EJC; EJC, excitatory junction current; GCLm, glutamate-cysteine ligase catalytic subunit M; GSNOR, S-nitrosoglutathione reductase; mEJC, miniature EJC; m5, muscle 5; m6, muscle 6; NO, nitric oxide; OE, overexpression; QC, quantal content.

Cpx acts by binding to the SNARE complex, thereby promoting the clamping of release, and only when replaced by synaptotagmin 1 in response to Ca^2+^ influx will vesicle fusion be initiated [14, 20]. *Dm*cpx function can be regulated by protein kinase A (PKA) phosphorylation of serine126 (Ser126) [7] or by prenylation at the C-terminus [15, 16]. In order to test whether cpx is required to exert NO effects, we first used *cpx* null mutants (*cpx*^*SH1*^, *cpx*^*-/-*^) [14]. In these animals, we detected a strong reduction in evoked release and QC (22.6 ± 3.2 [*n* = 11], *p* < 0.0001 versus Ctrl), which was unaffected by NO (13.8 ± 2.0 [*n* = 4], *p* > 0.05 versus *cpx*^*-/-*^, *p* < 0.0001 versus Ctrl, Fig 5A–5D and S5 Data). Similarly, when comparing the vesicle pool size, *cpx*^*-/-*^ NMJs showed a strong reduction (26 ± 6 [*n* = 11], *p* < 0.0001 versus Ctrl), which again was unaffected by NO (22 ± 5 [*n* = 4], *p* > 0.05 versus *cpx*^*-/-*^, *p* < 0.0001 versus Ctrl, Fig 5A–5D). These data confirm that cpx is required for NO to induce suppression of evoked release and available vesicle pool size and suggest that NO might enhance the clamping function of cpx in WT larvae. We next tested the impact of NO on the clamping ability of cpx by characterizing spontaneous release. Interestingly, the frequency of spontaneous events inversely correlates with endogenous cpx levels [14]. We analyzed mEJCs in *cpx*^*-/-*^ muscle 6 (m6), which exhibited an extremely high frequency [14] (>40 × *w*^*1118*^, Fig 5E). NO did not reduce the mEJC frequency in those preparations, although a precise analysis is difficult due to strong overlap of single mEJCs [14]. In order to allow more accurate frequency measurements in *cpx*^*-/-*^ animals, we used neighboring muscle 5 (m5), posessing a synapse with approximately 4-fold fewer release sites compared to m6. Similar to m6, *cpx*^*-/-*^ increased mEJC frequencies >10-fold compared to Ctrl (m5: *w*^*1118*^: 0.8 ± 0.2 s^−1^ [*n* = 3], *cpx*^*-/-*^: 11.6 ± 0.8 s^−1^ [*n* = 6], *p* < 0.0001); however, following NO exposure, this preparation did not show any change in mEJC frequency (m5 *cpx*^*-/-*^ + NO: 9.7 ± 0.9 s^−1^ [*n* = 5], *p* > 0.05 versus m5 *cpx*^*-/-*^, Fig 5E and 5F), suggesting the requirement of cpx for the observed nitrergic effects. Nevertheless, we recorded from m6 of heterozygous animals, which exhibit higher frequencies than *w*^*1118*^ but are still accurately quantifiable (m6 *cpx*^*+/-*^: 4.6 ± 0.8 s^−1^ [*n* = 5]). Here, NO induced a strong reduction in the frequency (m6 *cpx*^*+/-*^ + NO: 0.6 ± 0.2 s^−1^ [*n* = 5] #*p* < 0.05 versus m6 *cpx*^*+/-*^ Ctrl, Fig 5E and 5F), similar to that seen in *w*^*1118*^. These data confirm that NO only modulates spontaneous release frequencies in the presence of cpx.

Together, these data show that in the absence of cpx, NO causes no electrophysiological phenotypes. The NO-mediated reduction of eEJC amplitudes, QC, pool size, and mEJC frequency all require the presence of cpx, suggesting that its modulation might be responsible for the observed nitrergic effects, which could be explained by a gain-of-clamping function [53]. This potential effect was further investigated by using the established paradigm of activity-induced enhancement of spontaneous release at the *Drosophila* NMJ [7]. We assessed whether NO modulation of release also affects this activity-dependent signaling, which would strengthen the role of cpx as a target for nitrergic regulation and a general regulatory mechanism. PKA has been reported to modulate mEJC frequency potentiation in a cpx overexpression model (*Dm*cpx 7B, [7]). We confirmed that high frequency stimulation (50 Hz for 3 s) led to an enhanced mEJC frequency in *w*^*1118*^ NMJs relative to baseline (Ctrl: 1.9 ± 0.2-fold [*n* = 13], Fig 5G and 5H). Interestingly, repeating this protocol in larvae exposed to NO showed a lack of frequency potentiation (NO: 0.8 ± 0.1-fold [*n* = 14], *p* < 0.05 versus Ctrl), which was also ODQ independent (NO + ODQ: 1.0 ± 0.1-fold [*n* = 7], *p* > 0.05 versus NO, Fig 5G and 5H). To test whether the manipulation of PTMs also affects nitrergic suppression of frequency potentiation, we used larvae overexpressing GCLm and GSNOR and NOS “null” larvae. We found that GCLm and GSNOR overexpression occluded nitrergic effects on suppression of mEJC frequency potentiation, whereas the lack of NO signaling led to enhanced potentiation (GCLm + NO: 2.3 ± 0.4-fold [*n* = 7], GSNOR + NO: 2.5 ± 0.5-fold [*n* = 7], NOS “null” [comprised of *n* = 5 NOS^C^ and *n* = 3 NOS^Δ15^]: 3.8 ± 0.3-fold, **p < 0.01 versus Ctrl, ##*p* < 0.01 versus NO, ####*p* < 0.001 versus NO, Fig 5G and 5H). These data show that NO suppresses the activity-mediated increase in mEJC frequency and suggest that, similar to phospho-incompetent *cpx* mutants [7], nitrergic modulation of WT cpx produces an inhibitory action on spontaneous release. The lack of PTM signaling leads to an enhanced frequency potentiation, strengthening the notion that NO-mediated effects are responsible for suppression of synaptic release and our data point towards modulation of cpx as a key signaling mechanism.

### Nitrergic activity affects farnesylation of cpx and enhances its clamping properties

**Fig 6:**
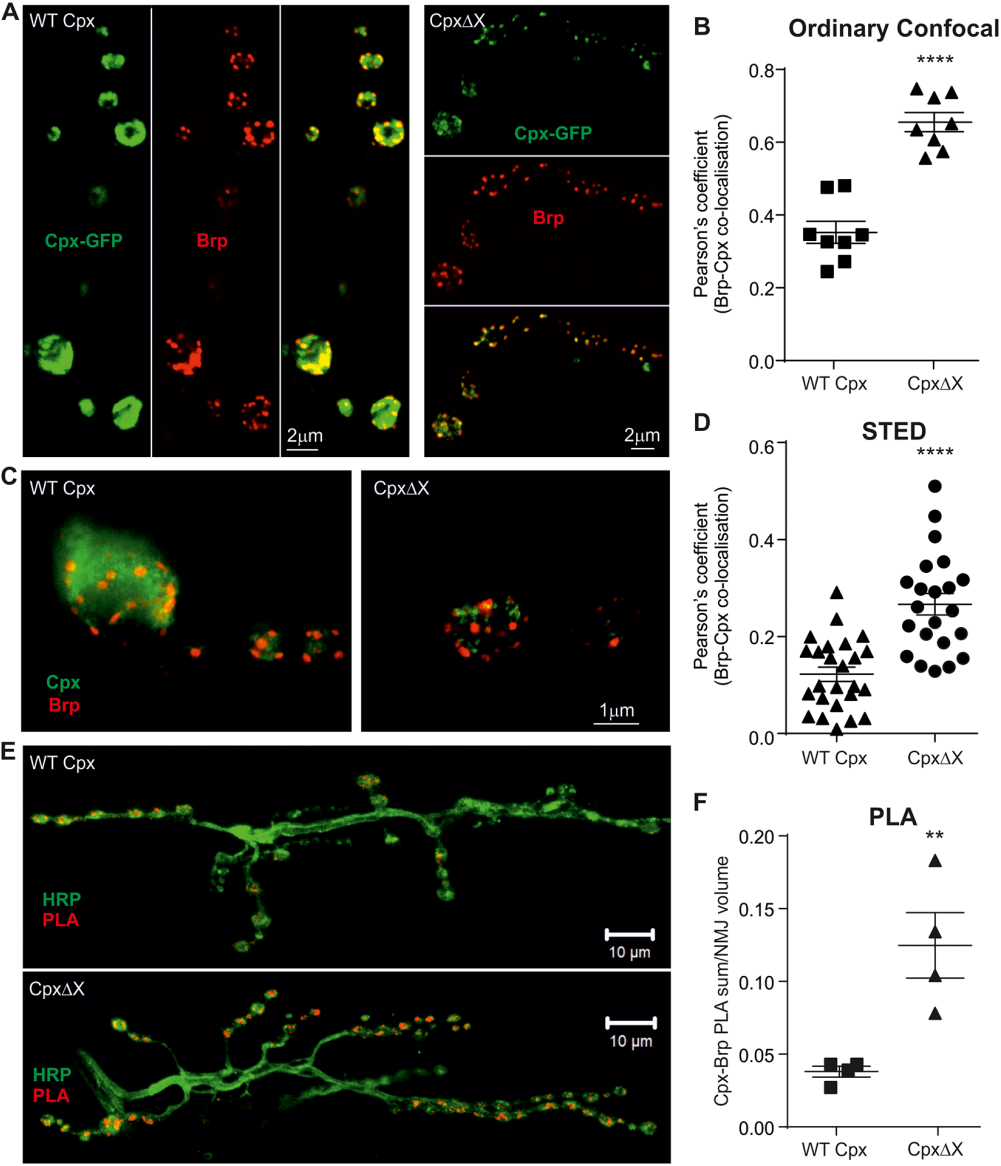
Lack of cpx farnesylation promotes its co-localization with AZs. (A) Representative maximal projection confocal images of GFP-tagged WT Cpx and mutant *Cpx*^*1257*^ (CpxΔX) at the NMJ (green: cpx, red: Brp). (B) Enhanced Cpx-Brp co-localization indicated by higher Pearson’s coefficient for CpxΔX (n–number of NMJs). (C) STED images showing cpx and Brp localization in single boutons in WT and CpxΔX mutants. The mutation (*Cpx*^*1257*^) increases the co-localization of cpx with Brp as indicated by the enhanced Pearson’s coefficient (D) (n–number of boutons). (E) Maximal projection confocal images of NMJs from larvae expressing WT Cpx and CpxΔX. PLA fluorescence shown in red and HRP staining in green. (F) Summated PLA signal volumes relative to NMJ volumes for cpx (GFP)-Brp interactions (n–number of NMJs). The raw data can be found in S6 Data. Data denote mean ± SEM, Student *t* test, **p = 0.009, ****p < 0.0001. AZ, active zone; Brp, Bruchpilot; cpx, complexin; GFP, green fluorescent protein; HRP, horseradish peroxidase; NMJ, neuromuscular junction; PLA, proximity ligation assay; STED, stimulated emission depletion; WT, wild-type.

Having shown that cpx signaling is involved in NO-mediated effects on spontaneous and evoked release, we next considered if S-nitrosylation of the Cys residue within the C-terminus of cpx possessing the CAAX motif could explain the observed results. Importantly, prenylation has been studied in several genetically modified cpx proteins in which the CAAX motif was eliminated [15, 16]. These studies suggest that deletion of final parts of the C-terminus/final amino acid affects cpx localization, interactions with SNARE-proteins, and, subsequently, its function. To explore the effects of cpx farnesylation more in detail, we made use of *Drosophila* lines expressing green fluorescent protein (GFP)-tagged WT and mutant cpx (*cpx*^*1257*^, lacking the final amino acid [16]), referred to as CpxΔX. This mutant has been shown to exhibit altered co-localization with syntaxin at the dorsolongitudinal flight muscle (DLM) neuromuscular synapse. We assessed localizations of WT and mutant cpx at the NMJ (*elav* > UAS-*cpx-GFP*, *elav* > *UAS-cpx*^*1257*^*-GFP*) with respect to their interaction with the AZ protein, Brp. WT cpx exhibits diffuse localization within boutons (as previously reported [15]) with little co-localization with Brp (Fig 6A and 6B and S6 Data). In contrast, the mutant form, lacking farnesylation, is highly co-localized with Brp, as indicated by the increase in Pearson’s coefficient (Fig 6A and 6B; WT cpx: 0.35 ± 0.30 [*n* = 9], CpxΔX: 0.65 ± 0.03 [*n* = 9], *p* < 0.0001). These data confirm that preventing cpx farnesylation results in enhanced co-localization with AZ. To further support these data, we conducted high-resolution stimulated emission depletion (STED) microscopy [54] and analyzed the Pearson’s coefficient for the co-localization of Brp with cpx. This experiment verified the confocal data showing enhanced co-localization of CpxΔX with Brp versus WT cpx (WT cpx: 0.13 ± 0.02 [*n* = 25], CpxΔX: 0.27 ± 0.02 [*n* = 23], *p* < 0.0001, Fig 6C and 6D). As *Dm*cpx possesses a predominant clamping function [23], we propose that NO could lead to a reduction in farnesylation, a consequent stronger interaction with the SNARE complex at the AZ, and thereby enhance its clamping function upon transmitter release. To specifically confirm co-localizations, we used the high-resolution proximity ligation assay (PLA), with which we imaged interactions of Brp with cpx. We used both lines, WT cpx-GFP and CpxΔX-GFP expressing larvae, and found that PLA signals are strongly enhanced at NMJs expressing the mutant cpx (Fig 6E and 6F; WT cpx: 0.04 ± 0.004 [*n* = 9], CpxΔX: 0.12 ± 0.02 [*n* = 9], *p* = 0.009). As the co-localization data may depend upon expression of GFP-tagged cpx, we confirmed equal GFP expression levels in both lines by immunoblotting (S9A Fig). These co-localization and PLA experiments confirm an enhanced association of a mutated farnesylation-incompetent cpx with Brp and suggest that lack of farnesylation renders cpx in close proximity to release sites of AZs.

In order to assess this possibility further, we used pharmacological and genetic tools to modulate cpx farnesylation and compared protein localization and synaptic release following farnesyl transferase (FTase) inhibition and NO exposure. Reduced expression of the *Drosophila* ortholog of FTase or inhibition of FTase by L-744,832 and GGTI-298 have strong effects on fly lethality [55], implicating a crucial function of this signaling in fly.

**Fig 7:**
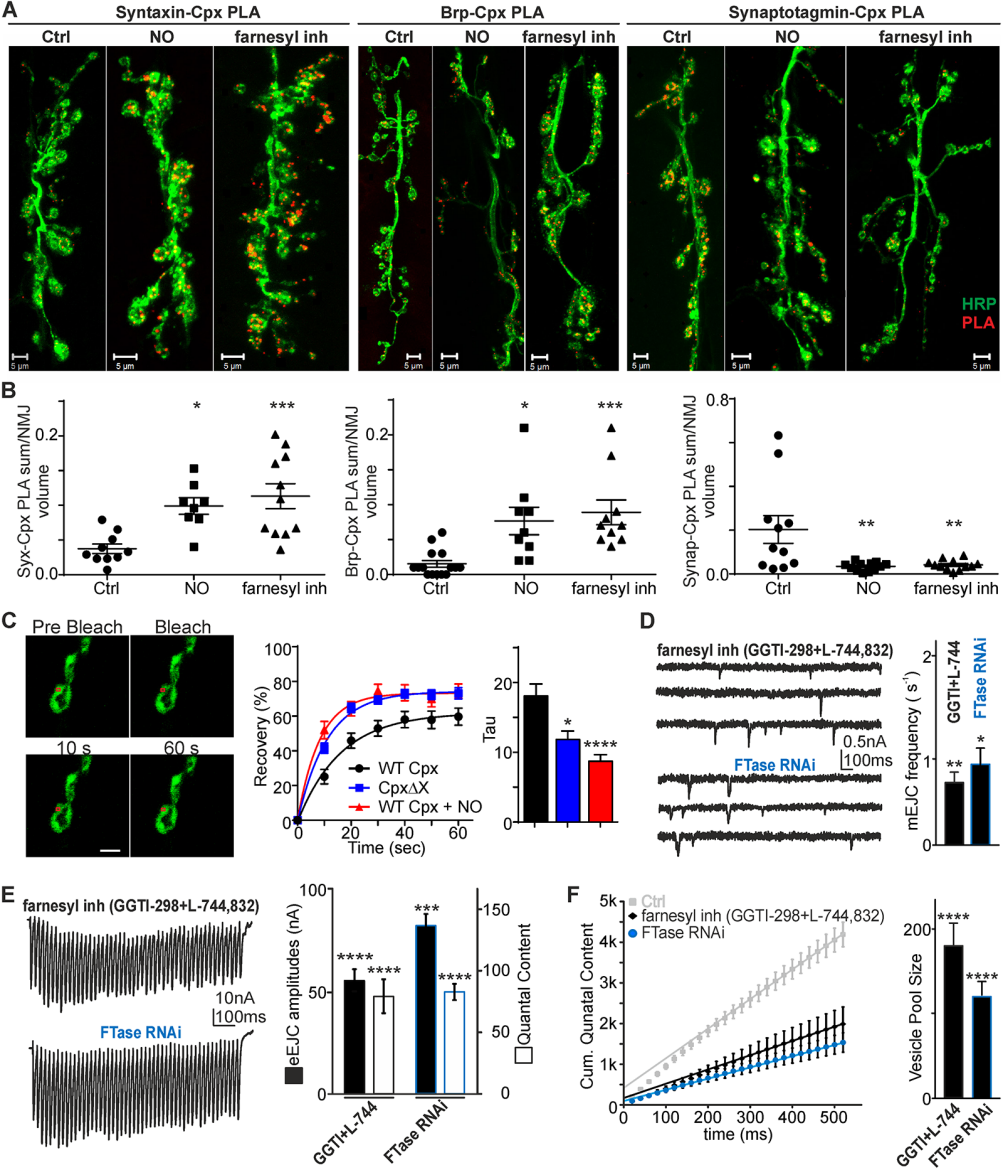
Reduced farnesylation of cpx enhances AZ localization and alters interactions with SNARE proteins. (A) Maximal projection confocal images of *w*^*1118*^ NMJs in Ctrl, following 60 min of exposure to NO or farnesylation inhibitor (“farnesyl inh”: 10 μM GGTI-298 + 20 μM L-744,832). PLA fluorescence in red and HRP staining in green for: left, syntaxin-cpx; middle: Brp-cpx; right: synaptotagmin-cpx interactions. (B) Analysis of summated PLA signal volumes relative to NMJ volumes. (C) FRAP experiments were performed at NMJs expressing GFP-cpx, shown as representative images of WT GFP-cpx at different time points (bleaching area: 2.5 μm^2^, scale bar: 2 μm). Right, mean data showing recovery of WT, CpxΔX, and NO-treated WT cpx, with mean tau values summarized. Note, lack of farnesylation due to the mutation or NO treatment results in faster recovery rates. (D) Representative mEJC recordings following 60 min incubation with GGTI-298 + L-744,832 (“farnesyl inh”) or of a larva expressing FTase-RNAi with mean mEJC frequencies. (E) Trains of 50-Hz stimulation of a larva incubated for 60 min with GGTI-298 + L-744,832 (“farnesyl inh”) or expressing FTase-RNAi with mean eEJC amplitudes and QC. (F) Cumulative QC of 50-Hz trains with mean estimated RRP sizes (right). The raw data can be found in S7 Data. Data denote mean ± SEM, Student *t* test, or ANOVA with post hoc Tukey-Kramer as indicated, *p < 0.05, **p < 0.01, ***p < 0.001, ****p < 0.0001. AZ, active zone; Brp, Bruchpilot; cpx, complexin; Ctrl; control; eEJC, evoked EJC; EJC, excitatory junction current; FRAP, fluorescence recovery after photobleaching; FTase, farnesyl transferase; GFP, green fluorescent protein; HRP, horseradish peroxidase; mEJC, miniature EJC; NMJ, neuromuscular junction; NO, nitric oxide; PLA, proximity ligation assay; QC, quantal content; RNAi, RNA interference; RRP, readily releasable pool; SNARE, soluble *N*-ethyl-maleimide-sensitive fusion protein Attachment Protein Receptor; WT, wild-type.

First, we tested how FTase inhibition (20 μM L-744,832 + 10 μM GGTI-298) and NO exposure affect cpx co-localization with the SNARE complex proteins syntaxin and synaptotagmin or Brp, using the PLA. We measured total PLA volume of NMJ z-stacks and normalized PLA signals to NMJ volume. We found that both treatments (depicted as “farnesyl inh” and “NO,” Fig 7A and 7B and S7 Data) led to enhanced co-localization of cpx with syntaxin and Brp (syntaxin-cpx: Ctrl: 0.04 ± 0.007, NO: 0.12 ± 0.02, farnesyl inh: 0.11 ± 0.02, Brp-cpx: 0.02 ± 0.007, NO: 0.08 ± 0.03, farnesyl inh: 0.09 ± 0.05, Fig 7A and 7B; *p* < 0.01, *p* < 0.001 versus Ctrl), suggesting that NO PTMs and farnesylation inhibition enrich cpx at the AZ. When analyzing the interactions between the Ca^2+^ sensor synaptotagmin and cpx, we found that this interaction was completely suppressed following treatments (Ctrl: 0.2 ± 0.06, NO: 0.03 ± 0.006, farnesyl inh: 0.04 ± 0.007, Fig 7A and 7B; *p* < 0.01 versus Ctrl). The PLA data were further supported by STED imaging studies showing identical changes in protein co-localization, as determined by Pearson’s coefficient analysis (S7 Fig and S9 Data). One possibility to allow for greater amounts of cpx to be available for binding to SNAREs is by enhancing free and soluble cytosolic levels as a consequence of reduced farnesylation. Farnesylation of cpx results in its membrane tethering, and thus protein fractions, which are membrane bound, are less mobile than soluble cytosolic proteins. To assess the mobility of potentially farnesylated versus soluble (non-farnesylated) cpx and thus distinguish between these two pools of cpx, we performed fluorescence recovery after photobleaching (FRAP) analysis of GFP-tagged WT and farnesylation-incompetent cpx (CpxΔX). Although a previous study did not detect differences between farnesylated versus non-farnesylated cpx isoform using this method with a photo-bleaching area of half a bouton [15], we found that accurate FRAP analysis of cpx-GFP mobility can only be performed by using substantially smaller bleaching areas, as reported previously [56] (S8 Fig and S9 Data). Using this approach, we found that bleaching an area of 2.5 μm^2^ (instead of >10 μm^2^) generally leads to faster recovery rates (S8 Fig and S9 Data). Our data confirmed that lack of farnesylation (CpxΔX) allows for greater movement of cpx and faster recovery (tau: WT cpx: 18.1 ± 1.7 ms, CpxΔX: 11.9 ± 1.2 ms [*p* < 0.05], WT Cpx + NO: 8.8 ± 0.8 ms [*p* < 0.0001], *n* = 18–20, Fig 7C), as expected for a soluble protein. Our data further show that NO treatment caused the same increase in recovery rates (Fig 7C), suggesting that NO also prevented farnesylation.

These data suggest that due to enriched local levels, cpx outcompetes synaptotagmin for SNARE binding at the AZ, thereby displacing synaptotagmin, as reported previously in biochemical studies [53]. Our data show that pharmacological and genetic inhibition of farnesylation promotes cpx co-localization with the AZ and supports the notion that this negatively impacts on synaptotagmin-SNARE complex binding, subsequently reducing release. The specificity of the PLA was corroborated by lack of Brp-cpx PLA signals in *cpx*^*-/-*^ larvae (S9B–S9D Fig). Next, we explored the possibility of whether specific inhibition of FTase activity by L-744,832 and GGTI-298 and FTase RNAi mimics the effects of NO on synaptic transmission. We found that, in both conditions, the frequency of mEJCs was reduced to similar values seen following NO exposure (f_mEJC_: L-744,832 + GGTI-298: 0.7 ± 0.1 s^−1^ [*n* = 8], *p* = 0.0051 versus Ctrl, FTase RNAi: 0.9 ± 0.2 s^−1^ [*n* = 9], *p* = 0.0136 versus Ctrl, Student *t* test, Fig 7D). Importantly, both L-744,832 + GGTI-298 and FTase RNAi expression reduced evoked transmission and available vesicle pool size to levels similar to those following NO incubation (L-744,832 + GGTI-298: eEJC: 56 ± 5 nA, QC: 80 ± 13 [*n* = 9], pool size: 180 ± 27 [*n* = 9], *p* < 0.0001 versus each *w*^*1118*^ Ctrl; FTase RNAi: eEJC: 75 ± 5 nA, QC: 82 ± 6 [*n* = 9], pool size: 120 ± 17 [*n* = 9], *p* < 0.0001 versus each *w*^*1118*^ Ctrl, Student *t* test, Fig 7E and 7F). These data suggest that the farnesylation status of cpx mediates nitrergic effects, resulting in changed SNARE protein interactions, which determines the physiological outcome of cpx.

**Fig 8:**
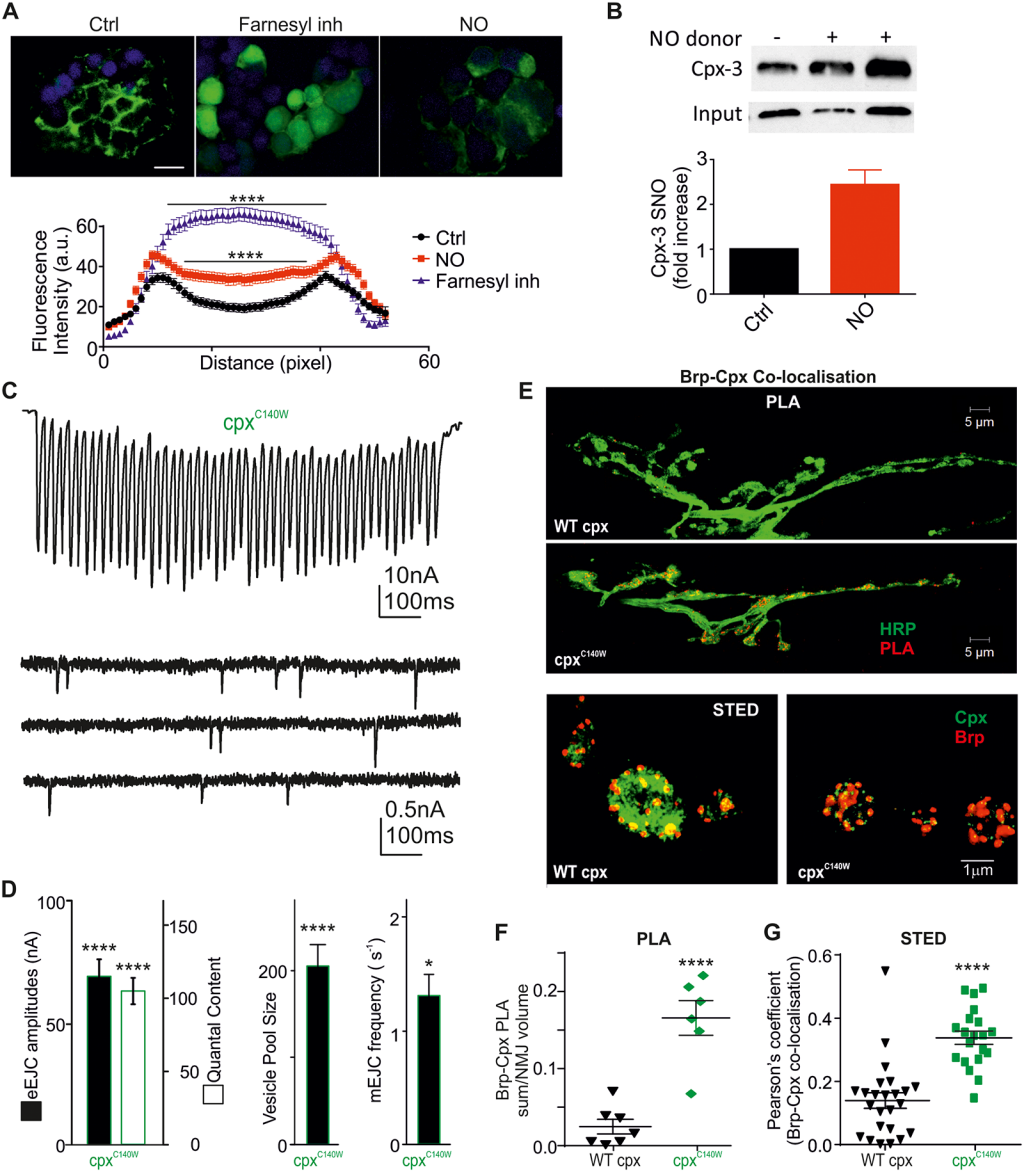
Cpx nitrosylation and block of farnesylation leads to redistribution of GFP-CAAX and enhances *Dm*cpx localization at AZs. (A) GFP-CAAX expression in HEK cells showing membrane fluorescence signals in Ctrls. Pharmacological inhibition of farnesylation by GGTI-298 + L-744,832 (“farnesyl inh,” *p* < 0.0001 versus Ctrl, ANOVA) and NO incubation (*p* < 0.0001 versus Ctrl, ANOVA) result in a redistribution of GFP fluorescence into the cytosol. Fluorescence signals were analyzed by line scan and plotted as intensities (a.u.) over distance across the cell somata, *n* = 80–104 cells, scale bar: 20 μm. (B) Mouse cpx-3 is S-nitrosylated in response to NO donor application. Immunoblot intensities increased 2.4 ± 0.5-fold following NO application. (C) Representative recordings of a 50-Hz train and spontaneous activity of a larva expressing *Dm*cpx 7A^C140W^ with mean eEJC amplitudes, QC, vesicle pool size, and mEJC frequency shown in (D). (E) Top, maximal projection confocal images of NMJs expressing a WT cpx or *Dm*cpx 7A^C140W^ showing the PLA signal in red; bottom, STED images showing cpx and Brp staining in WT and *Dm*cpx 7A^C140W^ mutants. (F) Analysis of PLA data. (G) Co-localization data with Pearson’s coefficient for interactions of cpx with Brp. The raw data can be found in S8 Data. Data denote mean ± SEM, Student *t* test (D, F), *p < 0.05, ***p < 0.001, ****p < 0.0001. a.u., arbitrary unit; AZ, active zone; Brp, Bruchpilot; cpx, complexin; Ctrl, control; eEJC, evoked EJC; EJC, excitatory junction current; GFP, green fluorescent protein; HEK, human embryonic kidney; HRP, horseradish peroxidase; mEJC, miniature EJC; NMJ, neuromuscular junction; NO, nitric oxide; PLA, proximity ligation assay; QC, quantal content; STED, stimulated emission depletion; WT, wild-type.

To further investigate the effects of NO directly on the prenylation process, we employed the well-characterized GFP-CAAX transfection model [57]. Here, human embryonic kidney (HEK) cells were transfected with GFP-CAAX (K-Ras motif) and the membrane association was assessed in response to prenylation inhibition and NO treatment. In control conditions, GFP exhibited a strong fluorescence signal at the membrane, which disappeared and redistributed into the cytosol following pharmacological inhibition of prenylation (L-744,832 + GGTI-298, *p* < 0.0001), confirming the prenylation-mediated localization of GFP-CAAX to the membrane (Fig 8A and S8 Data). Importantly, we showed that NO treatment (propylamine propylamine NONOate [PAPA-NONOate], *p* < 0.0001) induced a similar phenotype, with GFP being localized predominantly in a cytosolic manner—suggesting that NO prevents farnesylation through the same pathway (Fig 8A). To confirm that the Cys within the CAAX motif can undergo S-nitrosylation, we performed the Biotin Switch Assay on cpx-3 from isolated mouse retinas. NO donor incubation induced a >2-fold increase in SNO-cpx (Fig 8B), confirming this PTM on cpx and suggesting that this PTM is responsible for NO-induced changes in localization and function of cpx.

To specifically confirm the effects of S-nitrosylation and SNO interaction with farnesylation of cpx in *Drosophila*, we generated and expressed a nitroso-mimetic *cpx* mutant (*Dm*cpx 7A^C140W^) in a *cpx* null background (*cpx*^*SH1*^) and assessed synaptic responses. The Cys140 of *Dm*cpx is located within a hydrophobic region, as predicted in the Kyle Doolittle plot, which favors S-nitrosylation [58]. This mutant exhibits reduced evoked responses, QC, and vesicle pool sizes (eEJC: 70 ± 7 nA, QC: 106 ± 8, pool size: 204 ± 23 [*n* = 15 each], *p* < 0.0001 versus each *w*^*1118*^ Ctrl, Fig 8C and 8D), indicating that the mimicking of S-nitrosylation and simultaneous lack of farnesylation of cpx caused the observed changes. Importantly, this mutation also induced a reduction in spontaneous activity (f_mEJC_: 1.3 ± 0.2 s^−1^ [*n* = 15], *p* < 0.05 versus *w*^*1118*^ Ctrl, Fig 8C and 8D), reinforcing the argument of enhanced clamping function due to SNO formation and lack of farnesylation. The expression of WT cpx in the null background did not affect QC, pool size, or mEJC frequency (QC: 167 ± 17 [*n* = 5]; pool size: 381 ± 76 [*n* = 5]; f_mEJC_: 2.4 ± 0.4 s^−1^ [*n* = 10 each], *p* > 0.05 versus each *w*^*1118*^ Ctrl). To confirm changes in localization of *Dm*cpx 7A^C140W^, we analyzed PLA signals and found that *Dm*cpx 7A^C140W^ highly co-localizes with Brp, in strong contrast to WT cpx (WT: 0.025 ± 0.013, *Dm*cpx 7A^C140W^: 0.17 ± 0.03 [*n* = 6–7], *p* < 0.0001, both expressed in *cpx*^*-/-*^ background, Fig 8E and 8F). The data from the PLA experiments were confirmed by STED confocal microscopy, showing significantly higher Pearson’s coefficients for the co-localization of the cpx mutant C140W with Brp relative to the interaction of WT cpx with Brp (WT cpx: 0.13 ± 0.03, *Dm*cpx 7A^C140W^: 0.34 ± 0.02 [*n* = 20–24], *p* < 0.0001; Fig 8E and 8G). These data demonstrate that independent approaches to block farnesylation (and mimic of cpx-SNO) recapitulate nitrergic modulation of release and protein localization and therefore link for the first time NO-induced PTM and farnesylation signaling of cpx. We propose that S-nitrosylation acts as a novel endogenous pathway to alter cpx farnesylation signaling and protein–protein interactions and thereby allows a fine-tuning of synaptic function.

## Discussion

NO regulates a multitude of physiological and pathological pathways in neuronal function via generation of cGMP, thiol-nitrosylation, and 3-nitrotyrosination in health and disease [59]. Here, we show by employing biochemical and genetic tools in *Drosophila*, mouse, and HEK cells that NO can S-nitrosylate cpx and modulate—in a cGMP-independent manner—neurotransmitter release at the NMJ by interfering with its prenylation status, thereby affecting the localization and function of this fusion-clamp protein. We found that these nitrergic effects are reversed by GSH application or overexpression of GSH-liberating and de-nitrosylating enzymes (GCLm/c, GSNOR). GSH is the major endogenous scavenger for the NO moiety by the formation of S-nitrosoglutathione (GSNO) and consequently reduces protein-SNO levels via trans- and de-nitrosylation. The suppression of NOS activity facilitates synaptic function and the data support the notion that endogenous or exogenous NO enhances S-nitrosylation, reduces cpx farnesylation, and diminishes release.

Of the numerous synaptic molecules involved in release, cpx in particular has been implicated in the regulation of both evoked and spontaneous release due to its fusion-clamp activity. Despite the seemingly simple structure of cpx, its physiological function is highly controversial, as this small SNARE-complex binding protein can both facilitate but also diminish fast Ca^2+^-dependent and spontaneous release, depending on the system studied [22, 25, 53, 60]. In addition, there are different mammalian isoforms of cpx (1–4), which differ in their C-terminal region, with only cpx 3/4 containing the CAAX prenylation motif. Farnesylation in general determines protein membrane association and protein–protein interactions [61], and some cpx isoforms, such as *mus*cpx 3/4 and *Dm*cpx 7A, are regulated in this manner [13, 23, 62]. However, *mus*cpx 1/2 does not possess a CAAX motif, suggesting differential regulatory pathways to modulate cpx function. In *Drosophila*, there are alternative splice variants resulting from a single *cpx* gene, but the predominant isoform contains the CAAX motif (*Dm*cpx 7A), implicating the importance of this signaling molecule [15, 16]. The other splice isoform (*Dm*cpx 7B) lacks the CAAX motif and is expressed at about 1,000-fold lower levels at the larval stage [15], thus making *Dm*cpx 7A the dominant isoform to be regulated by farnesylation. However, the lack of *Dm*cpx 7B phosphorylation by PKA induces similar phenotypes as seen in our experiments when assessed following an induction of activity-dependent potentiation of mEJC frequency [7], which also may involve cpx–synaptotagmin 1 interactions.

Interestingly, both depletion and excessive levels of cpx suppress Ca^2+^-dependent and -independent exocytosis [63]. Cpx may promote SNARE complex assembly and simultaneously block completion of fusion by retaining it in a highly fusogenic state. Ca^2+^-dependent fusion is promoted below a concentration of 100 nM of cpx, whereas above 200 nM, it exhibits a clamping function resulting in a bell-shaped response curve [64]. Previous work suggests that synaptotagmin 1, once bound to Ca^2+^, relieves the cpx block and allows fusion. Another study reported that selective competition between cpx and synaptotagmin 1 for SNARE binding allows regulation of release [53]. Our data are in agreement with the latter findings, as we observed reduced synaptotagmin 1–cpx interactions following the block of farnesylation (Fig 7), indicating fewer synaptotagmin molecules binding to the SNARE complex to displace cpx. This limited replacement of cpx by synaptotagmin has been implicated in biochemical studies showing that local excess of cpx inhibits release, presumably by outcompeting synaptotagmin binding [53, 60]. Thus, synaptotagmin-SNARE binding is strongly dependent upon the local concentration of cpx [53]. Alternatively, and we cannot exclude this possibility, the modulation of cpx may simply alter its binding to the SNARE complex without directly displacing synaptotagmin, but interpretation of the data from our assays (PLA, co-localization) would not allow us to distinguish between these possibilities.

Our data are compatible with the idea that cpx binds to the SNARE complex, facilitates assembly, and then exerts its clamping function by preventing full fusion due to SNARE complex stabilization and subsequent increased energy barrier to allow fusion. Our model could provide an explanation of how cpx can be regulated to signal downstream to modulate transmitter release. So far, there are no data available, apart from mutation studies, as to how cpx function can be altered. We provide data indicating a physiologically relevant mechanism to adjust cpx function, possibly to the requirements of the neuron to adjust synaptic transmission. This likely occurs due to Cys S-nitrosylation and suppression of farnesylation, allowing greater amounts of hydrophilic cpx, not bound to endomembranes, to be available for binding with the SNARE complex in an altered configuration. This cross signaling between nitrosylation/farnesylation has been proposed to act as a molecular switch to modulate Ras activity [65]. Our data show that enhanced nitrergic activity and blocking farnesylation, either genetically (CpxΔX) or pharmacologically, alters the localization of cpx at the *Drosophila* NMJ and that of GFP-CAAX in HEK cells (Figs 6–8). Furthermore, by using a nitroso-mimetic *cpx* mutant, we found enhanced co-localization of cpx with the AZ protein Brp, implying a localization-function relationship (Fig 8). This consequently increases the net-clamping function because of elevated local concentrations of cpx. *Dm*cpx specifically exhibits a strong clamping function, as shown following overexpression in hippocampal neurons, which causes suppression of evoked and spontaneous release accompanied by a reduction of the release probability [23] or reduced vesicle fusion efficiency in in vitro assays [64].

Two independent studies eliminating the CAAX motif in *Dm*cpx (*cpx*^*572*^ and *cpx*^*1257*^) investigated localization-function interactions and showed disagreeing effects on both release and cpx localization [15, 16]. In particular, it has also been shown that the truncated cpx (*cpx*^*572*^, lacking the last 25 amino acids) does not co-localize with Brp [15]. Interestingly, this mutant causes a strong decrease in C-terminal hydrophobicity and a modest physiological response (increased mini frequency, decreased evoked amplitudes equivalent to a loss of clamping and loss of fusion function) relative to the total knock-out (KO). In contrast, the *cpx* mutant with single amino acid deletion (*cpx*^*1275*^) causes no effect on evoked but identical effects on the frequency of spontaneous release, suggesting a lack of clamping but no lack of fusion function. In addition, this mutant now co-localizes with the AZ at the NMJ [16]. These two studies indicate that the different mutations cause contrasting electrophysiological and morphological phenotypes, indicating that it is due to the nature of the mutation (lack of the last 25 amino acids versus 1 amino acid), which highlights the importance of a functional C-terminus. More recent studies have shown that deletions of the final amino acids (6 or 12 residues) completely abolished the membrane binding of cpx-1, impairing its inhibitory function and confirming the requirement of an intact C-terminus for inhibition of release [66, 67]. Here, we use an endogenous cpx with intact hydrophobic C-terminus, allowing physiological membrane binding. This is essential for inhibitory function, as the C-terminus is required for selective binding to highly curved membranes, such as those of vesicles [68]. Thus, as we used different approaches to alter farnesylation and generated a single amino acid mutant cpx (*Dm*cpx 7A^C140W^), leaving the C-terminus intact, our studies were performed under conditions of endogenous regulation of cpx function and thus provide new functional data on cpx signaling. Importantly, our data show that this regulation alters cpx function, and this is the first study to provide an explanation for the differential effects observed using *cpx* mutants or even cpx protein fragments in mammals, worm, and fly in various cross-species rescue experiments [20, 23].

Our data are in agreement with a model that non-farnesylated hydrophilic and soluble cytosolic cpx binds to the vesicular membrane via its C-terminal interactions, thereby exerting its inhibitory effect. When proteins are farnesylated, they are likely tethered to endomembranes, other than vesicle membranes [12]. It has to be distinguished between cpx interaction with the vesicle membrane as a result of the hydrophobic C-terminus, allowing cpx to become in close proximity to the AZ, and cpx endomembrane binding following farnesylation, which prevents cpx interactions with the AZ. However, in our case, SNO modification may enhance the binding to other proteins (e.g., SNAREs), thereby augmenting the effects. These additional interactions with unknown binding partners may affect proper cpx function and explain some of the discrepancies seen in studies using other genetically altered cpxs.

**Fig 9:**
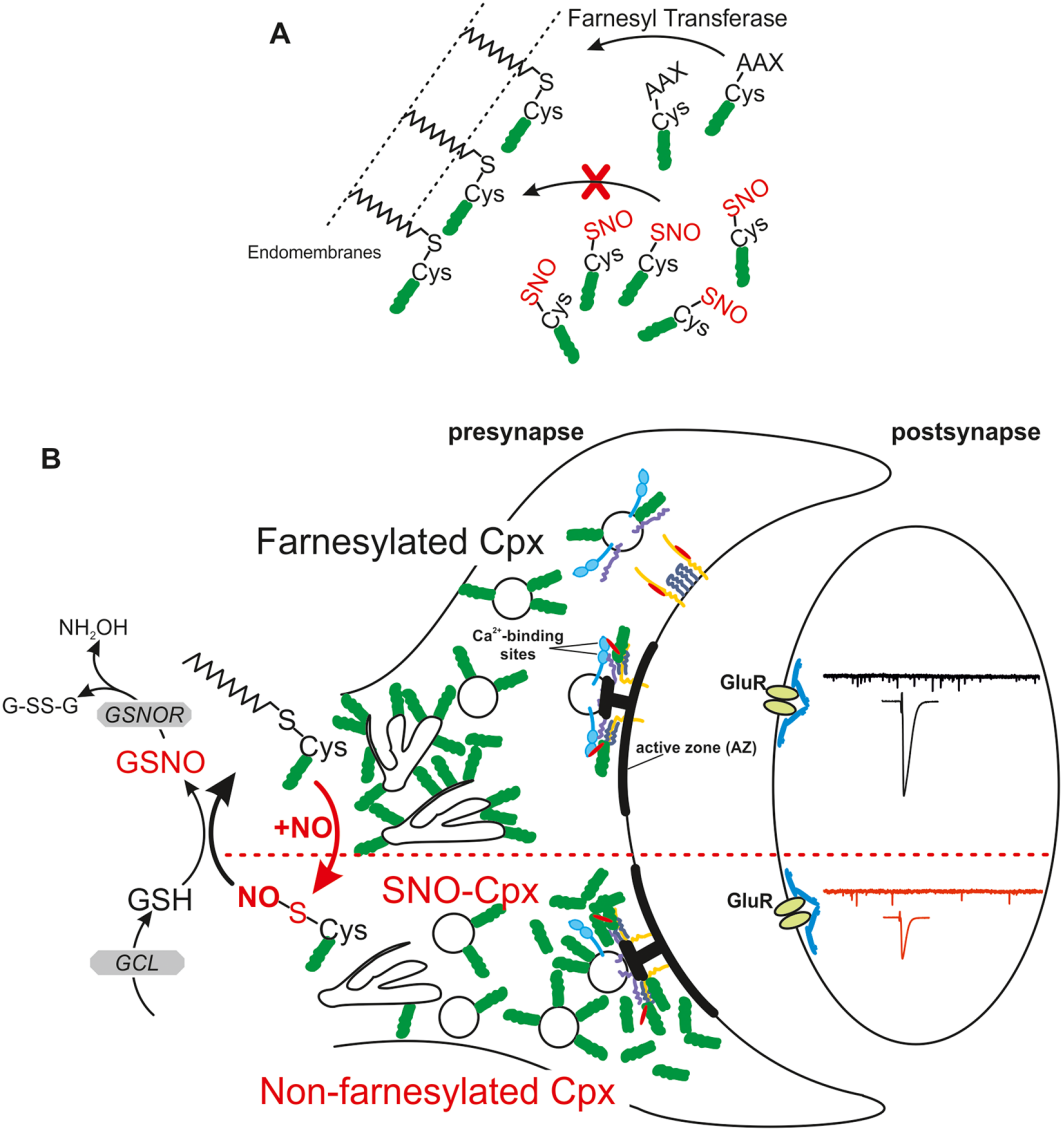
Effects of SNO formation on farnesylation and cpx function. (A) The farnesyl transferase facilitates the addition of a farnesyl group to cpx (green) containing the CAAX motif. Upon S-nitrosylation, this motif is not recognized and the protein will not be farnesylated, resulting in lack of endomembrane targeting. (B) The schematic shows the effects of S-nitrosylation of cpx Cys within CAAX, resulting in fewer proteins being farnesylated and tethered to endomembranes. This allows a greater proportion of cytosolic cpx to be able to bind to the SNARE complex (including syntaxin [yellow]/Munc-18 [red], SNAP-25 [dark purple], synaptobrevin [purple]) to compete with synaptotagmin [blue] binding and prevent fusion because of its clamping function, which results in reduced transmitter release. This process is reversible and depends on the availability of GSH-dependent de-nitrosylation of cpx. GSH is generated by GCL and GSNO is broken down into G-SS-G and NH_2_OH by GSNOR activity. Cys, cysteine; cpx, complexin; G-SS-G, glutathione disulphide; GCL, glutamate-cysteine ligase; GluR, glutamate receptor; GSH, glutathione; GSNO, S-nitrosoglutathione; GSNOR, S-nitrosoglutathione reductase; NH_2_OH, hydroxylamine; SNARE, soluble *N*-ethyl-maleimide-sensitive fusion protein Attachment Protein Receptor; SNO, S-nitrosothiol.

In summary, our study provides new data to illustrate a potential mechanism to regulate cpx function in a physiological environment, and we showed that NO acts as an endogenous signaling molecule that regulates synaptic membrane targeting of cpx, a pathway that may reconcile some of the controversial findings regarding cpx function. We suggest that increased S-nitrosylation and consequent lack of farnesylation leads to enhanced cytosolic levels of a soluble hydrophilic cpx and less endomembrane-bound fractions (Fig 9), because farnesylation-incompetent proteins remain in the cytosol [12]. These novel observations advance our understanding of similar nitrergic regulation of farnesylation that may be relevant for mammalian cpx-dependent synaptic transmission at the retina ribbon synapse and other brain regions [13]. Finally, this work has broader implications for physiological or pathological regulation of the prenylation pathway not only during neurodegeneration and aging, when enhanced S-nitrosylation might contribute to abnormal farnesylation signaling [69, 70], but also in other biological systems in which nitrergic activity and prenylation have important regulatory functions such as in cardio-vasculature or cancer signaling [71].

## Methods

### Fly stocks

Flies were raised on standard maize media at 25 °C at a 12-h LD cycle. The *elav*-Gal4 [C155] driver was obtained from the Bloomington Stock Center (Indiana, US). The UAS-RNAi lines (*GCLm* [CG4919], *GCLc* [CG2259], and *Fnta* [CG2976]) were purchased form the Vienna *Drosophila* Resource Centre (VDRC). The use of the UAS-Gal4 bipartite expression system to drive pan-neuronal expression excludes potential postsynaptic effects. The *elav*-Gal4 driver (female flies) and the UAS responder lines (male flies) were crossed to obtain offspring expressing the genes of interest and *w*^*1118*^ were used as Ctrls.

The fluorescent Ca^2+^ sensor GCaMP5 was tethered to the plasma membrane with an *N*-terminal myristoylation (myr) sequence as described previously [72]. The UAS-*myrGCaMP5* and *cpx*^*SH1*^ null mutant lines were provided by Troy Littleton (MIT, Cambridge, MA) [73]. GCaMP5 was expressed in glutamatergic neurons (OK371-Gal4; *UAS-GCaMP5*). cpx expression levels are shown for *w*^*1118*^ and *cpx*^*SH1*^ larvae in S9B and S9C Fig.

UAS-*fdh*31 (expression of fdh homologue of mammalian GSNOR/ADH-5) and UAS-*fdh*ri34/25 (expression of fdh RNAi) mutant transgenic lines were kindly provided by Li Liu Institute of Biophysics, Chinese Academy of Sciences, Beijing, China) [51].

NOS^Δ15^/NOS^C^ lines were provided by Patrick O’Farrell (UCSF, San Francisco, CA). *NOS*^*Δ15*^ deletion removes sequences encoding residues 1–757, encompassing the entire oxygenase domain and including regions that bind the catalytic heme and the substrate rendering the lines NOS “null” [33, 34].

The syn-null mutant transgenic line (*Syn*^*97*^) was generously provided by Erich Buchner (Universitätsklinikum Würzburg, Germany) [44]. UAS-*EGFP-cpx* and UAS-*EGFP-cpx1257* transgenic lines were kindly provided by Fumiko Kawasaki (Penn State University, PA) [16]. UAS-*GCLm* and UAS-*GCLc* transgenic lines were provided by William C. Orr (Southern Methodist University Dallas, TX).

### Cloning

cDNAs encoding for *cpx* 7A was a gift from Troy Littleton and used as a template for downstream PCRs. Cys 140 of *cpx7A* isoform was mutated to tryptophan to generate S-nitrosylation mimic mutant. PCR products, which include *XhoI* and *XbaI* restriction sites, were cloned into the pJFRC2 vector [74]—a gift from Gerald Rubin (Addgene plasmid no. 26214)—by standard methods. The resulting constructs were injected into attP40 *Drosophila* strains. The resulting transgenic lines (*Dmcpx7A*^*C140W*^ and WT *Dmcpx*) were crossed into a *cpx*^*SH1*^ background [7] using standard balancing techniques. The FlincG3 ORF was amplified from pTriEx4-H6-FGAm (FlincG3) (Addgene plasmid no. 49202) and the resultant PCR product cloned into pUASTattB by the Protein Expression Laboratory (PROTEX), University of Leicester. Microinjection of the pUASTattB plasmid was performed by the University of Cambridge, Department of Genetics Fly Facility.

### Electrophysiology

TEVC recordings were performed as described previously [75]. Sharp-electrode recordings were made from ventral longitudinal m6 in abdominal segments 2 and 3 of third instar larvae using pClamp 10, an Axoclamp 900A amplifier and Digidata 1440A (Molecular Devices, US) in hemolymph-like solution 3 (HL-3) [76]. Recording electrodes (20–50 MΩ) were filled with 3 M KCl. mEJCs were recorded in the presence of 0.5 μM tetrodotoxin (Tocris, UK). All synaptic responses were recorded from muscles with input resistances ≥4 MΩ, holding currents <4 nA at −60 mV and resting potentials more negative than −60 mV at 25 °C, as differences in recording temperature cause changes in glutamate receptor kinetics and amplitudes [77]. Holding potentials were −60 mV. The extracellular HL-3 contained (in mM): 70 NaCl, 5 KCl, 20 MgCl_2_, 10 NaHCO_3_, 115 sucrose, 5 trehalose, 5 HEPES, and 1.5 CaCl_2_ (0.5–3.0 mM in Fig 3 and S3 Data, as specified). Average single eEJC amplitudes (stimulus: 0.1 ms, 1–5 V) are based on the mean peak eEJC amplitude in response to 10 presynaptic stimuli (recorded at 0.2 Hz). Nerve stimulation was performed with an isolated stimulator (DS2A, Digitimer). Paired-pulse experiments were performed by applying 5 repetitive stimuli (0.2 Hz) at different intervals (20, 40, 100, 200 ms) for each cell at each ISI. All data were digitized at 10 kHz and for miniature recordings, 200-s recordings, we analyzed to obtain mean mEJC amplitudes, decay, and frequency (f) values. QC was estimated for each recording by calculating the ratio of eEJC amplitude/average mEJC amplitude, followed by averaging recordings across all NMJs for a given genotype. mEJC and eEJC recordings were off-line low-pass filtered at 500 Hz and 1 kHz, respectively. Materials were purchased from Sigma-Aldrich (UK) unless otherwise stated.

### Variance-mean analysis of eEJCs

Approximately 40 eEJCs were elicited at different [Ca^2+^]_e_, ranging from 0.5 to 3 mM to give mean eEJC amplitudes (I). The mean eEJC is given by I = Np_vr_q [45], with *N* being the number of independent release-ready vesicles, *p*_*vr*_ the vesicular release probability, and *q* the quantal size at each given [Ca^2+^]_e_. The eEJC variance was calculated as previously described [45]. The plots of the variance-mean were obtained for each cell and fitted with the parabolic function Var(I) = I^2^/N + qI that was a constraint to pass through the origin. Upon fitting the parabola, *p*_*vr*_ and *q* were calculated using the equations: *q* = A/(1+CV^2^) and *p*_*vr*_ = I(B/A)(1+CV^2^) where CV^2^ is the coefficient of variation of the eEJC amplitudes at a given [Ca^2+^]_e_ concentration calculated as CV^2^ = (eEJCs standard deviation/mean amplitude)^2^; A and B were obtained from the fitting parameters. Estimated values were not corrected for variability in mEJC amplitude distributions or latency fluctuations.

Ca^2+^ cooperativity was assessed by plotting eEJC amplitudes over [Ca^2+^]_e_ and fitted with the Hill equation (mean eEJC amplitude plotted versus different [Ca^2+^]_e_: eEJC([Ca^2+^]) = eEJC_max_[1+(EC_50_/[Ca^2+^])^slope^]^−1^), yielding the Hill slope as a measure of Ca^2+^ cooperativity.

### Cumulative postsynaptic current analysis

The apparent size of the RRP was probed by the method of cumulative eEJC amplitudes [78]. Muscles were clamped to −60 mV and eEJC amplitudes during a stimulus train (50 Hz, 500 ms [of a 1-s train]) were calculated as the difference between peak and baseline before stimulus onset of a given eEJC. Receptor desensitization was not blocked as it did not affect eEJC amplitudes, because a comparison of the decay of the first and the last eEJC within a train did not reveal any significant difference in decay kinetics. The number of release-ready vesicles (*N*) was obtained by back extrapolating a line fit to the linear phase of the 500-ms cumulative eEJC plot (the last 200 ms of the train) to time zero. *N* was then obtained by dividing the cumulative eEJC amplitude at time zero by the mean mEJC amplitude recorded in the same cell. To calculate the QC in the train, we used mean mEJC amplitudes measured before the train.

### Immunohistochemistry

Third instar larvae were dissected in ice-cold PBS then fixed in 4% paraformaldehyde. After permeabilization with PBS-0.1% Triton (PBS-T) and blocking with PBS-T containing 0.2% bovine serum albumin (BSA) and 2% normal goat serum, larval fillets were incubated at 4 °C overnight in solutions of primary antibody. The following antibody dilutions were used: NC82 (supernatant) anti-Brp (Bruchpilot) 1:200, cpx (1:500), syntaxin (1:200), synaptotagmin (1:200), and GFP (1:200). After 3 × 10 min washes in PBS-T, larvae were incubated with AlexaFluor 488 goat anti-HRP (Jackson Immuno Research) and AlexaFluor 546 goat anti-mouse 1:500 dilution for 90 min at room temperature. Larvae were mounted using Vectashield mounting medium (Vector Labs) and NMJ 6/7 (segments A2 and A3) images were acquired with a Zeiss laser-scanning confocal microscope (LSM 510, Zeiss). Image analysis was performed with ZEN (Zeiss) and Volocity 6.3 software.

### STED microscopy

Images were acquired on a Leica TCS SP8 system attached to a Leica DMi8 inverted microscope (*Leica Microsystems*). Excitation light (488 nm for AlexaFluor488 or 561 nm for AlexaFluor568) was provided by a white light laser with a repetition rate of 80 MHz. Images were acquired using a 100× 1.4 NA oil immersion objective and fluorescence was detected through a bandpass of 495–550 nm (AlexaFluor488 detection) or 570–650 nm (AlexaFluor 561 detection). Gated STED imaging of samples was achieved through use of 592-nm and 660-nm depletion lasers with a time gate set to 1.8–8 ns using the Leica STED 3X system. All images were acquired with 32-line averages and 22 × 22 nm pixel size.

### FRAP imaging

Images were taken using an LSM 510 confocal microscope (Zeiss). The size of the bleaching area was optimized as shown previously [56]. Bleaching areas were selected within each bouton (about 2.5 μm^2^) and images acquired every 10 s. Data were fitted with a single exponential to reveal tau values of fluorescence recoveries.

### PLA

The assay was performed as described [79]. Briefly, dissected third instar larvae were fixed in Bouin’s solution for 15 mins on ice, washed in PBT (PBS with 0.1% Triton) 3 times for 10 min each and blocked in PBT/1% BSA for 1 h. Larvae were incubated overnight at 4 °C in mouse and rabbit antibodies against the 2 proteins of interest, diluted in PBT/1% BSA. Primary antibodies used were anti-rabbit cpx (Littleton), anti-rabbit GFP (Abcam), anti-mouse Brp (Developmental Studies Hybridoma Bank [DSHB]), anti-mouse syntaxin (DSHB), and anti-mouse Synaptotagmin (DSHB). All antibodies were used at 1:200 dilution. The next day, PLA probe binding, ligation, and amplification steps were performed as described [79]. Before mounting, larvae were counterstained with AlexaFluor 488 goat anti-HRP (Jackson Immuno Research) at 1:500 dilution for 40 mins. PLA signals were only measured within the HRP signals. PLA signal and NMJ volumes of z-stack images were analyzed in Volocity 6.3. PLA signals were only measured within the HRP signals. All PLA signals were expressed relative to total NMJ volume (S10 Fig and S9 Data).

### HEK cell transfection

A plasma membrane targeted eYFP CAAX protein was constructed by fusing the last 15 amino acids of Human K-Ras isoform b with the C-terminus of eYFP. A short linker sequence GTMASNNTASG was inserted between the last amino acid of eYFP and the membrane targeting CAAX sequence. The resulting construct was subcloned into expression vector pcDNA5 frt and verified by DNA sequencing.

HEK293 FT cells were plated on poly-d-lysine coated glass coverslips in 6 well plates and transfected with 0.5 g eYFP CAAX per well using polyethylenimine (PEI) at a ratio of 1 g DNA to 6 g PEI. Prior to imaging, cells were treated for 12 h with the NO donor DETA-NONOate or a combination of the farnesyl transferase inhibitor L-744,832 (20 μM) and the geranylgeranyltransferase I inhibitor GGTI-298 (20 μM). Cells were then washed 3 times with PBS and fixed for 15 min with 4% paraformaldehyde. Coverslips were mounted on glass microscope slides with VectaShield H1500 and observed using a Zeiss laser scanning confocal microscope.

### Biotin switch assay

Animals were kept in the dark 3 h before removing the retinas in order to decrease basal levels of protein nitrosylation. Retinas were kept in DMEM (Gibco 31053–028) with protease inhibitors (Complete) and treated with NO donors (GSNO and PAPA-NONOate, 20 μM) for 40 min at room temperature and protected from light. The biotin-switch assay was performed with the S-nitrosylated Protein Detection kit (Cayman Chemical, 10006518) in the dark. Bradford assay was performed and equal amount of proteins were incubated with Streptavidin beads (Sigma) overnight. Western blot was performed with cpx 3 antibody (Synaptic Systems), 1:1,000.

### Electron microscopy

Third instar larvae were “filleted” in phosphate-buffered saline at room temperature and then fixed in 2% (wt/vol) glutaraldehyde in 0.1 M sodium cacodylate buffer (pH 7.4) at 4 °C overnight. They were postfixed with 1% (wt/vol) osmium tetroxide/1% (wt/vol) potassium ferrocyanide for 1 h at room temperature and then stained en bloc, overnight, with 5% (wt/vol) aqueous uranyl acetate at 4 °C, dehydrated and embedded in Taab epoxy resin (Taab Laboratories Equipment Ltd, Aldermaston, UK). Semi-thin sections, stained with toluidine blue, were used to identify areas containing synaptic regions (m6/7 in regions A2/A3). Ultra-thin sections were cut from these areas, counterstained with lead citrate, and examined in an FEI Talos transmission electron microscope (FEI Company [Thermo Fisher Scientific Inc.], Hillsboro, OR). Images were recorded using an FEI Ceta-16M CCD camera with 4k × 4k pixels. SV measurements were made using ImageJ software. A total of about 500–900 SVs were measured in 5–10 boutons from 3 animals per genotype.

### GCaMP imaging

Wandering third instar larvae expressing presynaptic UAS-*myrGCaMP5* or UAS-*GCaMP5* using the pan-neuronal C155 or glutamatergic neuronal OK371 driver, respectively, were dissected in low Ca^2+^ HL-3 saline (0.2 mM CaCl_2_) at room temperature. The motor nerves were carefully snipped below the ventral nerve cord, and the CNS was removed. The preparation was washed several times with HL-3 containing 1.5 mM Ca^2+^. Nerve stimulation was performed with an isolated stimulator (DS2A, Digitimer) and images were recorded before, during (2–6 s in a train at 60 Hz) and after the stimulation period (8 s) in HL-3 containing 3 mM Ca^2+^ or during 15 s in a 20-s train at 20 Hz at indicated Ca^2+^ levels in the presence of 5 mM L-glutamic acid. We acquired images at a rate of 1 image per 4 s using a Zeiss laser-scanning confocal microscope (LSM 510 Meta; Zeiss) with a 63× 1.0 NA water immersion objective (Zeiss). Excitation was set at 488 nm (Argon laser) using a dichroic mirror 490 nm and a bandpass filter 500–550 nm. Low sampling rates were sufficient to investigate Ca^2+^ plateau levels during the 8-s stimulation periods [80]. A single confocal plane of muscle pair 6/7 NMJ in segments A2 or A3 was imaged to establish a baseline. Small *z*-drifts were manually corrected during the imaging session. Imaging sessions in which significant movement of the muscle occurred were discarded. Images were analyzed using Volocity 6.3 Image Analysis software (PerkinElmer). Single bouton fluorescence intensities were measured (average within a bouton) and bouton ΔF/F_0_ values were averaged for each NMJ.

### FlincG3 imaging

NMJs of larvae expressing UAS-*FlincG3* presynaptically were imaged as described above to measure GCaMP fluorescence. To prevent cGMP breakdown by PDE activity, preparations were incubated with 10μM Zap prior to imaging.

### Mitochondrial respiratory activity assay

High resolution respirometry was performed with an Oroboros O2K oxygraph (Oroboros Instruments Ltd.). For each measurement, 3 third instar larvae were homogenized in 100 μL of respiration buffer MiR05 [81]. Leak state respiration was measured after adding 5 mM of pyruvate, 2 mM of malate, and 10 mM of glutamate. Oxphos capacity supported by Complex I was measured after addition of 1.25 mM ADP. After addition of 10 mM succinate, Oxphos capacity supported by both Complex I and Complex II were measured. Free Oxphos capacity was calculated as the difference Oxphos–Leak. Respiratory Ctrl ratios (RCRs) were calculated as the ratio Oxphos/Leak.

### cGMP radio immunoassay

Larval brains (30 per condition) were isolated and assessed for cGMP production. Briefly, brain extracts were diluted 5-fold in 100 mM sodium acetate, pH 6.2, and acetylated by consecutive addition of triethylamine (10 μL) and acetic anhydride (5 μL) and used in the radioimmunoassay [82] within 60 min. Cyclic GMP standards (100 μL; 0–4 nM) were treated identically. Acetylated samples (100 μL) were mixed with 2′-*O*-succinyl 3-[^125^I]-iodotyrosine methyl ester cyclic GMP (GE Healthcare, IM107) (50 μL, about 3,000 d.p.m. made up in 50 mM sodium acetate, 0.2% BSA, pH 6.2), and 100 μL of anti-cyclic GMP antibody (GE Healthcare, TRK500; diluted in 50 mM sodium acetate, 0.2% BSA, pH 6.2). Samples were intermittently vortex mixed during a 4-h incubation at 4 °C. Free and bound cyclic GMP was separated by charcoal precipitation with 500 μL of a charcoal suspension (1% [w/v] activated charcoal in 100 mM potassium phosphate, 0.2% BSA, pH 6.2). After vortex mixing for 5 min, samples were centrifuged (13,000 × *g*, 4 min, 4 °C) and radioactivity determined in an aliquot of supernatant (600 μL). Unknown values were determined from the cyclic GMP standard curve using GraphPad Prism 7 (GraphPad Software Inc., San Diego, CA). Data points represent 2 measurements of 30 brains for each condition.

### Drug applications

NO donor solutions were made freshly from stock solutions on the day and working solutions (200 μM sodium nitroprusside [SNP] and 5 μM PAPA-NONOate, each releasing about 200 nM NO) [29] were kept on ice for up to 6 h. All experiments to assess NO signaling were made between 40 and 60 min of NO exposure (NO: 200 μM SNP, 20 μM PAPA-NONOate; presented data comprise responses following incubation with either donor as they are not different from each other [Student *t* test, *p* > 0.05]; 500 μM SNP was used in Figs 7A, 7B, 8E and 8F [S7 and S8 Data]). Incubations with drugs: Zap (PDE inhibitor), ODQ (sGC inhibitor), L-744,832, and GGTI-298 (FTase inhibitors) incubation for 1 h; both block FTase with an IC_50_: 1.8 nM and IC_50_: 203 nM, respectively [83]. Drugs were purchased from Tocris or Sigma.

### Statistics

Statistical analysis was performed with Prism 6.3 and 7 and InStat 3 (Graphpad Software Inc., San Diego, CA). Statistical tests were carried out using an ANOVA test when applicable with a posteriori test (1-way ANOVA with Tukey’s multiple comparisons test) or unpaired Student *t* test, as indicated. Data are expressed as mean ± SEM where *n* is the number of boutons, NMJs, or larvae as indicated and significance is shown as *p < 0.05, **p < 0.01, ***p < 0.001, and ****p < 0.0001.

## Supporting information

S1 Fig Activities of mitochondrial complex I and II are not affected by NO signaling. Larval preparations were incubated either in Ctrl HL-3 or HL-3 + NO donors for 50 min and mitochondrial function was assessed by high resolution respirometry using an Oroboros Oxygraph-2K. (A) Oxygen fluxes (pmol/(s*ml) of Ctrl [red] and NO-treated [green] larvae; arrows below indicate additions of the following substrates: pyruvate, malate, glutamate, and succinate. (B) Summary of O_2_ flux measurements, left (Complex I: Ctrl: 23 ± 5, NO: 23 ± 3, Complex I+II: Ctrl: 32 ± 7, NO: 32 ± 3). Right, respiratory control ratios: oxidative phosphorylation/leak (Complex I: Ctrl: 10 ± 1, NO: 9 ± 2, Complex I+II: Ctrl: 14 ± 1 NO: 13 ± 2). The raw data for this figure can be found in S9 Data. Data denote mean ± SEM, *p* > 0.05, ANOVA with post hoc Tukey-Kramer was used for comparisons: Ctrl versus NO, *n* = 6 larvae each. ADP, adenosine diphosphate; Ctrl, control; D, ADP; G, glutamate; M, malate; NO, nitric oxide; PYR, pyruvate; S, succinate.
(TIF)

S2 Fig NOS null background does not cause developmental effects on NMJ size and ultrastructure (related to Fig 2). (A) NMJ volume was calculated from z-stack confocal images (HRP) in both genotypes (NMJ volume: WT: 3,183 ± 287 μm^3^ [*n* = 46 NMJs], NOS^Δ15^: 3,178 ± 284 μm^3^ [*n* = 11 NMJs], NOS^C^: 3,282 ± 494 μm^3^ [*n* = 5 NMJs], *p* > 0.05, ANOVA; Brp puncta/NMJ volume: WT: 0.07 ± 0.01 [*n* = 6 NMJs], NOS^Δ15^: 0.11 ± 0.01 [*n* = 7 NMJs], NOS^C^: 0.11 ± 0.01 [*n* = 5 NMJs], *p* > 0.05, ANOVA with post hoc Tukey-Kramer was used for comparisons). (C) Representative electron microscopy images of 1b boutons from each genotype, with red semicircles indicating the area for vesicle counts. (D) Mean values for the number of synapses (AZ), number of T-bars and number of vesicles within a 250-nm semicircle radius from the center of the AZ for each genotype (number of synapses: WT: 3.8 ± 0.3, NOS^C^: 4.2 ± 0.5; number of T-bars: WT: 1.2 ± 0.1, NOS^C^: 1.7 ± 0.3, number of vesicles: WT: 23 ± 1, NOS^C^: 25 ± 1 [*n* = 24 and *n* = 16 boutons per genotype], *p* > 0.05 for all comparisons, Student *t* test). The raw data for this figure can be found in S9 Data. Data denote mean ± SEM. AZ, active zone; Brp, Bruchpilot; HRP, horseradish peroxidase; NMJ, neuromuscular junction; WT, wild-type.
(TIF)

S3 Fig Recovery from depletion is not affected by NO signaling. (A) Recordings showing a 1-s 50-Hz train of eEJCs with subsequent single eEJCs at various time points. Note the broken trace to accommodate for the long intervals. (B) Mean eEJC amplitudes at increasing intervals after high-frequency stimulation (50Hz, 1s) for Ctrl (black, *n* = 16 NMJs), NO (red, *n* = 15 NMJs) and NO+ODQ (orange, *n* = 7 NMJs) with single exponential fits to the data points. (C) Mean time constant for recovery tau values for the conditions indicated (9.6 ± 1.2 s [Ctrl], 10.4 ± 1.1 s [NO], and 9.4 ± 1.9 s [NO + ODQ]). The raw data for this figure can be found in S9 Data. Data denote mean ± SEM, *p* > 0.05; ANOVA with post hoc Tukey-Kramer was used for comparisons. Ctrl, control; eEJC, evoked EJC; EJC, excitatory junction current; NMJ, neuromuscular junction; NO, nitric oxide; ODQ, 1H-[1,2,4]oxadiazolo[4,3-a]quinoxalin-1-one.
(TIF)

S4 Fig NO supresses evoked release and vesicle pools in syn knock-out NMJs. (A) Representative 50Hz trains of synaptic stimuli in Ctrl syn null mutant (*Syn*^*97*^ Ctrl) and NO-treated syn null mutant (*Syn*^*97*^ + NO) NMJs. (B) Cumulative QC for both conditions, showing the reduced available pool size following NO incubation. (C) Mean QC and vesicle pool sizes for conditions indicated (QC: 157 ± 20, NO: 84 ± 16; pool size: Ctrl: 288 ± 39, NO: 134 ± 23). The raw data for this figure can be found in S9 Data. Data denote mean ± SEM, *p < 0.05 versus *w*^*1118*^ Ctrl, ^#^*p* < 0.05 versus its Ctrl, ^&&^*p* < 0.01 versus its Ctrl, ANOVA for each comparing QC and vesicle size, *n* = 5 NMJs each. Ctrl, control; NMJ, neuromuscular junction; NO, nitric oxide; QC, quantal content; syn, synapsin.
(TIF)

S5 Fig Activity-induced intracellular Ca^2+^ levels are not affected by NO (related to Fig 3). (A) Confocal images of GCaMP5 expressing NMJs. GCaMP5 was expressed in motor neurons and fluorescence was imaged during a train of synaptic stimulation at 20 Hz. Experiments were performed at various extracellular Ca^2+^ concentrations (0.25–1.5 mM, as indicated) in Ctrl (representative images) and NO-treated NMJs. (B) Summary of ΔF/F_0_ for conditions indicated. Mean ΔF/F_0_: 0.25 Ca^2+^: Ctrl: 0.24 ± 0.04, NO: 0.14 ± 0.03, 0.5 Ca^2+^: Ctrl: 0.42 ± 0.09, NO: 0.5 ± 0.07, 1.5 Ca^2+^: Ctrl: 1.1 ± 0.1, NO: 1.2 ± 0.2. NO does not affect the presynaptic Ca^2+^ levels at any concentration tested. *p* > 0.05, ANOVA with post hoc Tukey-Kramer was used for comparisons. *n* = 7–11 NMJs per condition. The raw data for this figure can be found in S9 Data. Ctrl, control; NMJ, neuromuscular junction; NO, nitric oxide.
(TIF)

S6 Fig Reduction of endogenous de-nitrosylation capacity partially supresses synaptic release (related to Fig 4). (A) Mean eEJC amplitudes (GSNOR RNAi: Ctrl: 95 ± 10 nA, NO: 85 ± 7 nA, GCLm RNAi: Ctrl: 118 ± 11 nA, NO: 78 ± 4 nA). (B) QC (GSNOR RNAi: Ctrl: 170 ± 8, NO: 139 ± 10, GCLm RNAi: Ctrl: 129 ± 17, NO: 97 ± 12) and (C) vesicle pool sizes estimated by back extrapolation from cumulative QCs (GSNOR RNAi: Ctrl: 223 ± 38, NO: 214 ± 48, GCLm RNAi: Ctrl: 228 ± 38, NO: 98 ± 24), all for genotypes indicated. (E) Western blot analysis of GSNOR (*fdh*31, *RNAi-fdh*25) expression in genotypes indicated. The raw data for this figure can be found in S9 Data. Data denote mean ± SEM, *p < 0.05, **p < 0.01, ***p < 0.001, ****p < 0.0001 versus *w*^*1118*^ Ctrl, ^#^*p* < 0.05 versus its Ctrl, ANOVA with post hoc Tukey-Kramer was used for comparisons, *n* = 10–8 NMJs. Ctrl, control; eEJC, evoked EJC; EJC, excitatory junction current; *fdh*, formaldehyde dehydrogenase; GCLm, glutamate-cysteine ligase modifier subunit M; GSNOR, S-nitrosoglutathione reductase; NMJ, neuromuscular junction; NO, nitric oxide; QC, quantal content; RNAi, RNA interference.
(TIF)

S7 Fig Nitrergic activity alters cpx localization relative to SNARE and Brp proteins (related to Fig 7). (A) Representative STED confocal images of boutons from Ctrl larvae and those exposed to NO donor. (B) Co-localization analysis reveals Pearson’s coefficients for indicated conditions (Cpx-Brp: Ctrl: 0.24 ± 0.04, NO: 0.37 ± 0.02, farnesyl inh: 0.47 ± 0.02, Cpx-Syx: Ctrl: 0.12 ± 0.04, NO: 0.26 ± 0.03, farnesyl inh: 0.30 ± 0.04, Cpx-Synap: Ctrl: 0.42 ± 0.04, NO: 0.18 ± 0.03, farnesyl inh: 0.18 ± 0.04), *n* = 8–30 boutons; data denote mean ± SEM, *p < 0.05, **p < 0.01, ***p < 0.001, ****p < 0.0001. ANOVA with post hoc Tukey-Kramer was used for comparisons. The raw data for this figure can be found in S9 Data. Brp, Bruchpilot; cpx, complexin; Ctrl, conrol; NO, nitric oxide; SNARE, soluble *N*-ethyl-maleimide-sensitive fusion protein Attachment Protein Receptor; STED, stimulated emission depletion; Synap, synaptotagmin; Syx, syntaxin.
(TIF)

S8 Fig FRAP analysis reveals differences in recovery depending on the bleaching area (related to Fig 7). (A) Representative images of boutons expressing WT cpx-GFP before bleaching and at different time points after photo bleaching. Top row shows recordings with a bleaching area roughly the size of half a bouton (>10 μm^2^); bottom row shows images using a bleaching area of 2.5 μm^2^. (B) Analysis of recovery from bleach shows faster time constants using the smaller bleaching area compared to half-bouton bleach. Using the smaller bleaching areas, there is a pronounced difference between WT and mutant cpx. Synaptobrevin was used as a control for photo bleaching associated with vesicular movement. Note, images and analysis for the 2.5 μm^2^ bleaching areas are the same as in Fig 7C; scale bar: 2 μm. The raw data for this figure can be found in S9 Data. cpx, complexin; FRAP, fluorescence recovery after photobleaching; GFP, green fluorescent protein; WT, wild-type.
(TIF)

S9 Fig Confirmation of cpx expression. (A) Western blot analysis shows expression levels of cpx, GFP, and β-actin in *w*^*1118*^, WT cpx-GFP and cpxΔX-GFP lines. (B) Representative images of Brp-Cpx PLA in 2 example *cpx*^*SH1*^ NMJs show no PLA signal. (C) Confocal single plane images of a *w*^*1118*^ NMJ stained for cpx and Brp and (D) confocal single plane image of a *cpx*^*SH1*^ larva that does not express cpx. Brp, Bruchpilot; cpx, complexin; GFP, green fluorescent protein; NMJ, neuromuscular junction; PLA, proximity ligation assay; WT, wild-type.
(TIF)

S10 Fig NMJ volumes are not different across genotypes. NMJs for different genotypes and conditions have similar volumes compared to Ctrls (WT Cpx-GFP [Cpx^2A^]: 2,857 ± 261 μm^3^, CpxΔX-GFP [Cpx^1257^]: 3,395 ± 787 μm^3^, NO treatment: 3,400 ± 228 μm^3^, farnesyl inh: 3,973 ± 306 μm^3^, Cpx WT: 3,617 ± 875 μm^3^, Cpx^C140W^: 3,508 ± 707 μm^3^). Data denote mean ± SEM; ANOVA with post hoc Tukey-Kramer was used for comparisons versus *w*^*1118*^ WT, *p* > 0.05. The raw data for this figure can be found in S9 Data. Cpx, complexin; Ctrl, control; GFP, green fluorescent protein; NMJ, neuromuscular junction; NO, nitric oxide; WT, wild-type.
(TIF)

S1 Data Raw values used to generate graphs in Fig 1. The raw data presented in worksheets 1–5 serve as underlying data for Fig 1A–1F and 1G.
(XLSX)

S2 Data Raw values used to generate graphs in Fig 2. The raw data presented in worksheets 1–4 serve as underlying data for Fig 2B–2D and 2F–2H.
(XLSX)

S3 Data Raw values used to generate graphs in Fig 3. The raw data presented in worksheets 1–5 serve as underlying data for Fig 3C–3E, 3G and 3I.
(XLSX)

S4 Data Raw values used to generate graphs in Fig 4. The raw data presented in worksheets 1–4 serve as underlying data for Fig 4A–4F.
(XLSX)

S5 Data Raw values used to generate graphs in Fig 5. The raw data presented in worksheets 1–2 serve as underlying data for Fig 5B–5D and 5F.
(XLSX)

S6 Data Raw values used to generate graphs in Fig 6. The raw data presented in worksheets 1–3 serve as underlying data for Fig 6B, 6D and 6F.
(XLSX)

S7 Data Raw values used to generate graphs in Fig 7. The raw data presented in worksheets 1–5 serve as underlying data for Fig 7B–7F.
(XLSX)

S8 Data Raw values used to generate graphs in Fig 8. The raw data presented in worksheets 1–5 serve as underlying data for Fig 8A, 8B, 8D, 8F and 8G.
(XLSX)

S9 Data Raw values used to generate graphs in S1–S8 and S10 Figs. The raw data presented in worksheets 1–9 serve as underlying data for S1–S8 and S10 Figs.
(XLSX)

## Abbreviations

ADH-5: alcohol dehydrogenase 5
a.u.: arbitrary unit
AZ: active zone
Brp: Bruchpilot
BSA: bovine serum albumin
[Ca^2+^]_e_: extracellular calcium concentration
cGMP: cyclic guanosine monophosphate
CNS: central nervous system
cpx: complexin
Ctrl: control
Cys: cysteine
DLM: dorsal longitudinal flight muscles
DSHB: Developmental Studies Hybridoma Bank
EC_50_: half maximal effective concentration
eEJC: evoked EJC
EJC: excitatory junction current
EM: electron microscopy
ER: endoplasmic reticulum
*fdh*: formaldehyde dehydrogenase
FRAP: fluorescence recovery after photobleaching
FTase: farnesyl transferase
GCL: glutamate-cysteine ligase
GFP: green fluorescent protein
GluR: gluatamte receptor
GSH: glutathione
GSNO: S-nitrosoglutathione
GSNOR: S-nitrosoglutathione reductase
G-SS-G: glutathione disulphide
HRP: horseradish peroxidase
HL-3: hemolymph-like solution 3
ISI: interspike interval
KO: knock out
m5: muscle 5
m6: muscle 6
mEJC: miniature EJC
myr: *N*-myristoylation
*N*: number of release-ready vesicles
NH_2_OH: hydroxylamine
NMJ: neuromuscular junction
NO: nitric oxide
NOS: nitric oxide synthase
ODQ: 1H-[1,2,4]oxadiazolo[4,3-a]quinoxalin-1-one
PAPA-NONOate: propylamine propylamine NONOate
PBS-T: PBS-0.1% Triton
PDE: phosphodiesterase
PEI: polyethylenimine
PKA: protein kinase A
PLA: proximity ligation assay
PPR: paired pulse ratio
PROTEX: Protein Expression Laboratory
PTM: posttranslational modification
*p*_*vr*_: presynaptic release probability
*q*: quantal size
QC: quantal content
RCR: respiratory Ctrl ratio
RNAi: RNA interference
RP: reserve pool
RRP: readily releasable pool
sGC: soluble guanylyl cyclase
SNARE: soluble *N*-ethyl-maleimide-sensitive fusion protein Attachment Protein Receptor
SNO: S-nitrosothiol
SNP: sodium nitroprusside
STED: stimulated emission depletion
syn: synapsin
VDRC: Vienna *Drosophila* Resource Centre
WT: wild-type
Zap: zaprinast
