# Supplementary figures and images for: Nitric oxide-mediated posttranslational modifications control neurotransmitter release by modulating complexin farnesylation and enhancing its clamping ability
Source: PLoS Biol. 2018 Apr 9;16(4):e2003611. doi: 10.1371/journal.pbio.2003611 (PMC5890968; doi:10.1371/journal.pbio.2003611)

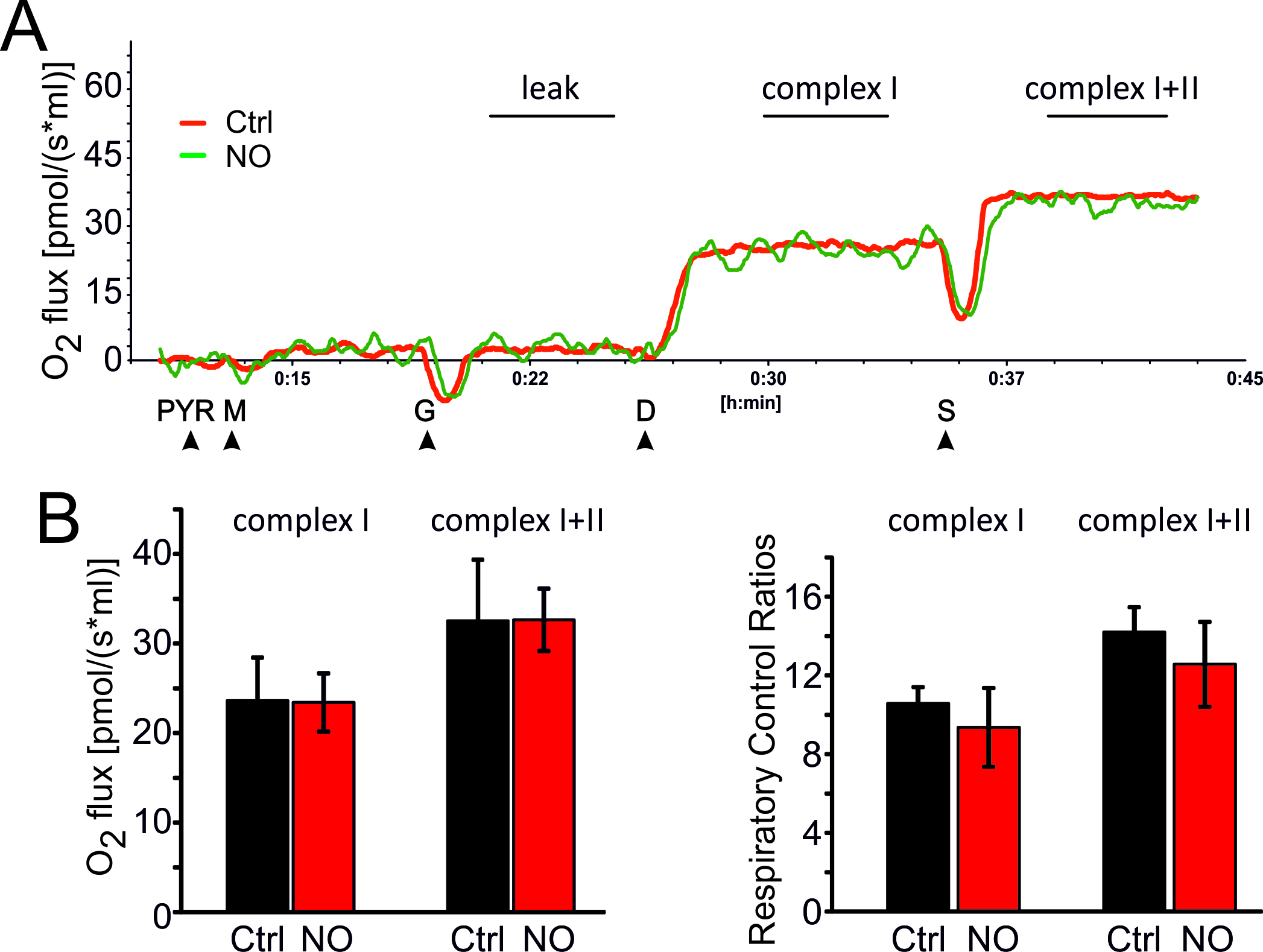

Supplement: S1 Fig — Larval preparations were incubated either in Ctrl HL-3 or HL-3 + NO donors for 50 min and mitochondrial function was assessed by high resolution respirometry using an Oroboros Oxygraph-2K. (A) Oxygen fluxes (pmol/(s*ml) of Ctrl [red] and NO-treated [green] larvae; arrows below indicate additions of the following substrates: pyruvate, malate, glutamate, and succinate. (B) Summary of O2 flux measurements, left (Complex I: Ctrl: 23 ± 5, NO: 23 ± 3, Complex I+II: Ctrl: 32 ± 7, NO: 32 ± 3). Right, respiratory control ratios: oxidative phosphorylation/leak (Complex I: Ctrl: 10 ± 1, NO: 9 ± 2, Complex I+II: Ctrl: 14 ± 1 NO: 13 ± 2). The raw data for this figure can be found in S9 Data. Data denote mean ± SEM, p > 0.05, ANOVA with post hoc Tukey-Kramer was used for comparisons: Ctrl versus NO, n = 6 larvae each. ADP, adenosine diphosphate; Ctrl, control; D, ADP; G, glutamate; M, malate; NO, nitric oxide; PYR, pyruvate; S, succinate. (TIF) [file pbio.2003611.s001.tif]

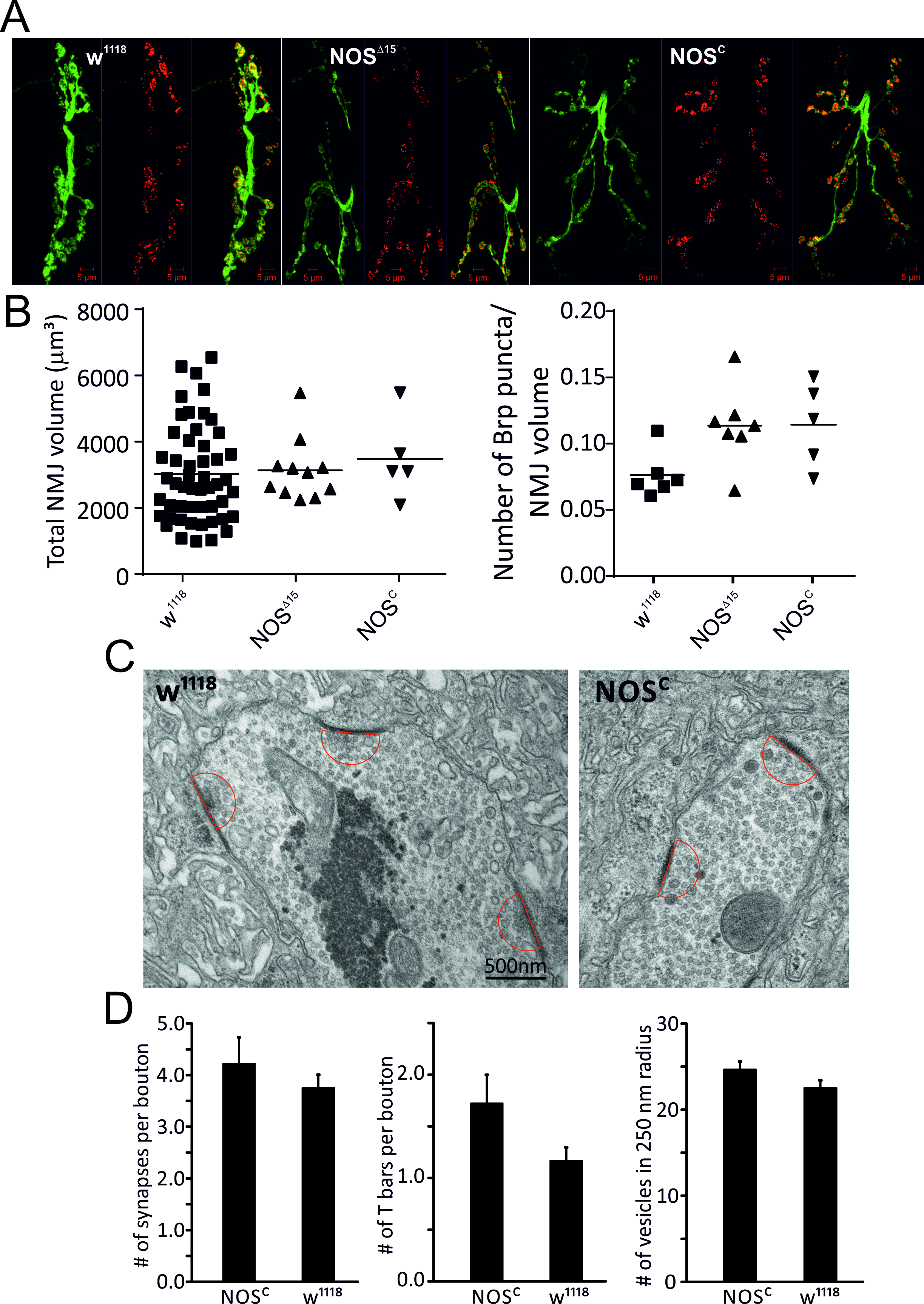

Supplement: S2 Fig — (A) NMJ volume was calculated from z-stack confocal images (HRP) in both genotypes (NMJ volume: WT: 3,183 ± 287 μm3 [n = 46 NMJs], NOSΔ15: 3,178 ± 284 μm3 [n = 11 NMJs], NOSC: 3,282 ± 494 μm3 [n = 5 NMJs], p > 0.05, ANOVA; Brp puncta/NMJ volume: WT: 0.07 ± 0.01 [n = 6 NMJs], NOSΔ15: 0.11 ± 0.01 [n = 7 NMJs], NOSC: 0.11 ± 0.01 [n = 5 NMJs], p > 0.05, ANOVA with post hoc Tukey-Kramer was used for comparisons). (C) Representative electron microscopy images of 1b boutons from each genotype, with red semicircles indicating the area for vesicle counts. (D) Mean values for the number of synapses (AZ), number of T-bars and number of vesicles within a 250-nm semicircle radius from the center of the AZ for each genotype (number of synapses: WT: 3.8 ± 0.3, NOSC: 4.2 ± 0.5; number of T-bars: WT: 1.2 ± 0.1, NOSC: 1.7 ± 0.3, number of vesicles: WT: 23 ± 1, NOSC: 25 ± 1 [n = 24 and n = 16 boutons per genotype], p > 0.05 for all comparisons, Student t test). The raw data for this figure can be found in S9 Data. Data denote mean ± SEM. AZ, active zone; Brp, Bruchpilot; HRP, horseradish peroxidase; NMJ, neuromuscular junction; WT, wild-type. (TIF) [file pbio.2003611.s002.tif]

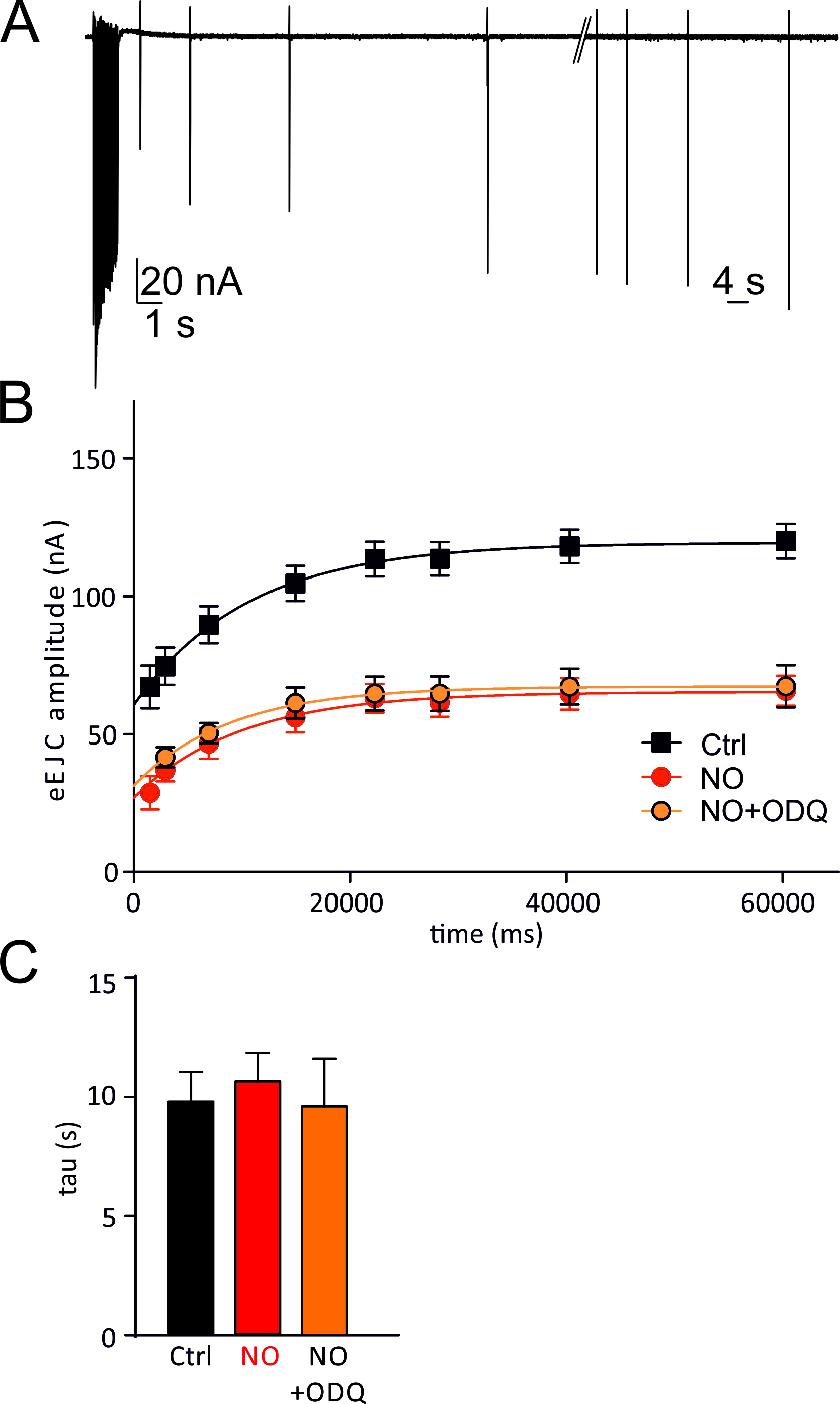

Supplement: S3 Fig — (A) Recordings showing a 1-s 50-Hz train of eEJCs with subsequent single eEJCs at various time points. Note the broken trace to accommodate for the long intervals. (B) Mean eEJC amplitudes at increasing intervals after high-frequency stimulation (50Hz, 1s) for Ctrl (black, n = 16 NMJs), NO (red, n = 15 NMJs) and NO+ODQ (orange, n = 7 NMJs) with single exponential fits to the data points. (C) Mean time constant for recovery tau values for the conditions indicated (9.6 ± 1.2 s [Ctrl], 10.4 ± 1.1 s [NO], and 9.4 ± 1.9 s [NO + ODQ]). The raw data for this figure can be found in S9 Data. Data denote mean ± SEM, p > 0.05; ANOVA with post hoc Tukey-Kramer was used for comparisons. Ctrl, control; eEJC, evoked EJC; EJC, excitatory junction current; NMJ, neuromuscular junction; NO, nitric oxide; ODQ, 1H-[1,2,4]oxadiazolo[4,3-a]quinoxalin-1-one. (TIF) [file pbio.2003611.s003.tif]

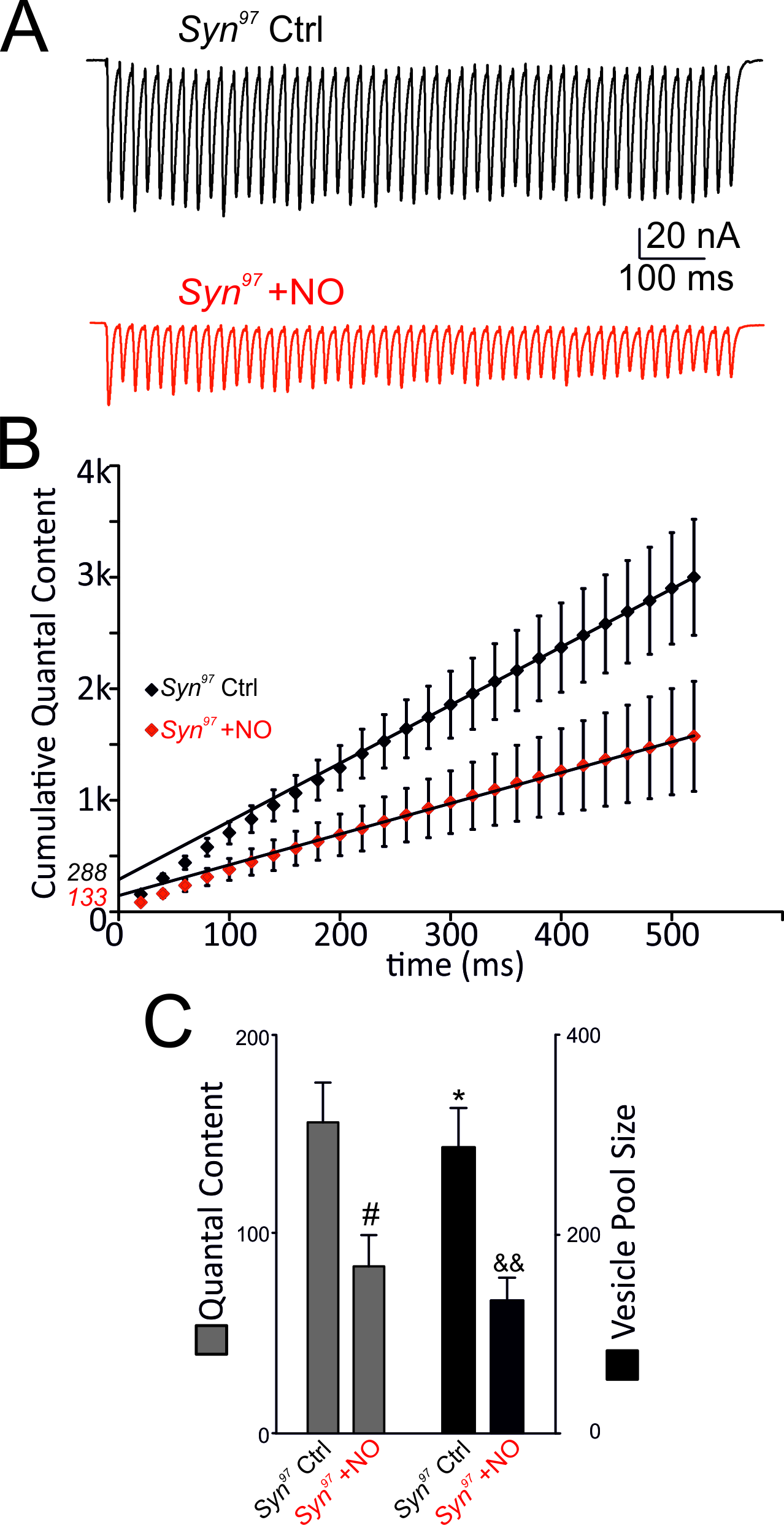

Supplement: S4 Fig — (A) Representative 50Hz trains of synaptic stimuli in Ctrl syn null mutant (Syn97 Ctrl) and NO-treated syn null mutant (Syn97 + NO) NMJs. (B) Cumulative QC for both conditions, showing the reduced available pool size following NO incubation. (C) Mean QC and vesicle pool sizes for conditions indicated (QC: 157 ± 20, NO: 84 ± 16; pool size: Ctrl: 288 ± 39, NO: 134 ± 23). The raw data for this figure can be found in S9 Data. Data denote mean ± SEM, *p < 0.05 versus w1118 Ctrl, #p < 0.05 versus its Ctrl, &&p < 0.01 versus its Ctrl, ANOVA for each comparing QC and vesicle size, n = 5 NMJs each. Ctrl, control; NMJ, neuromuscular junction; NO, nitric oxide; QC, quantal content; syn, synapsin. (TIF) [file pbio.2003611.s004.tif]

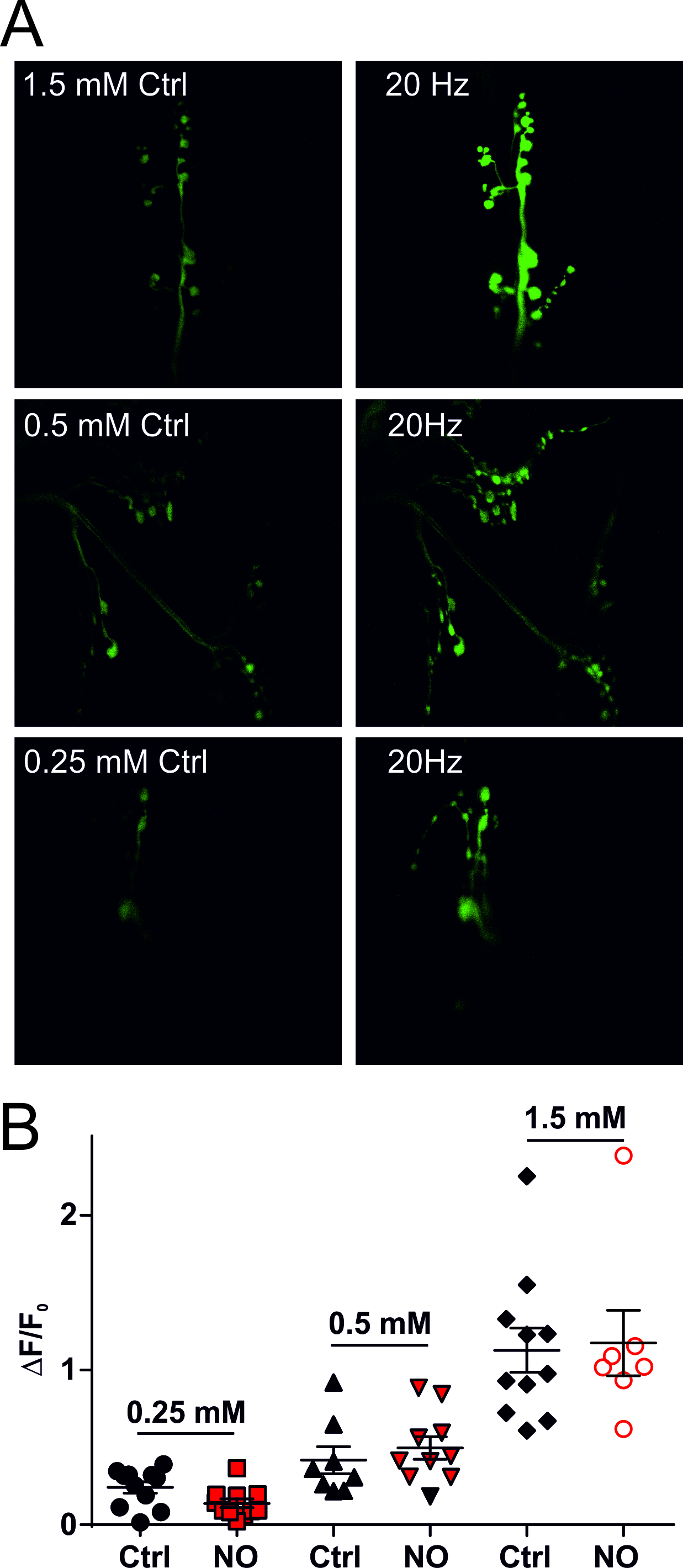

Supplement: S5 Fig — (A) Confocal images of GCaMP5 expressing NMJs. GCaMP5 was expressed in motor neurons and fluorescence was imaged during a train of synaptic stimulation at 20 Hz. Experiments were performed at various extracellular Ca2+ concentrations (0.25–1.5 mM, as indicated) in Ctrl (representative images) and NO-treated NMJs. (B) Summary of ΔF/F0 for conditions indicated. Mean ΔF/F0: 0.25 Ca2+: Ctrl: 0.24 ± 0.04, NO: 0.14 ± 0.03, 0.5 Ca2+: Ctrl: 0.42 ± 0.09, NO: 0.5 ± 0.07, 1.5 Ca2+: Ctrl: 1.1 ± 0.1, NO: 1.2 ± 0.2. NO does not affect the presynaptic Ca2+ levels at any concentration tested. p > 0.05, ANOVA with post hoc Tukey-Kramer was used for comparisons. n = 7–11 NMJs per condition. The raw data for this figure can be found in S9 Data. Ctrl, control; NMJ, neuromuscular junction; NO, nitric oxide. (TIF) [file pbio.2003611.s005.tif]

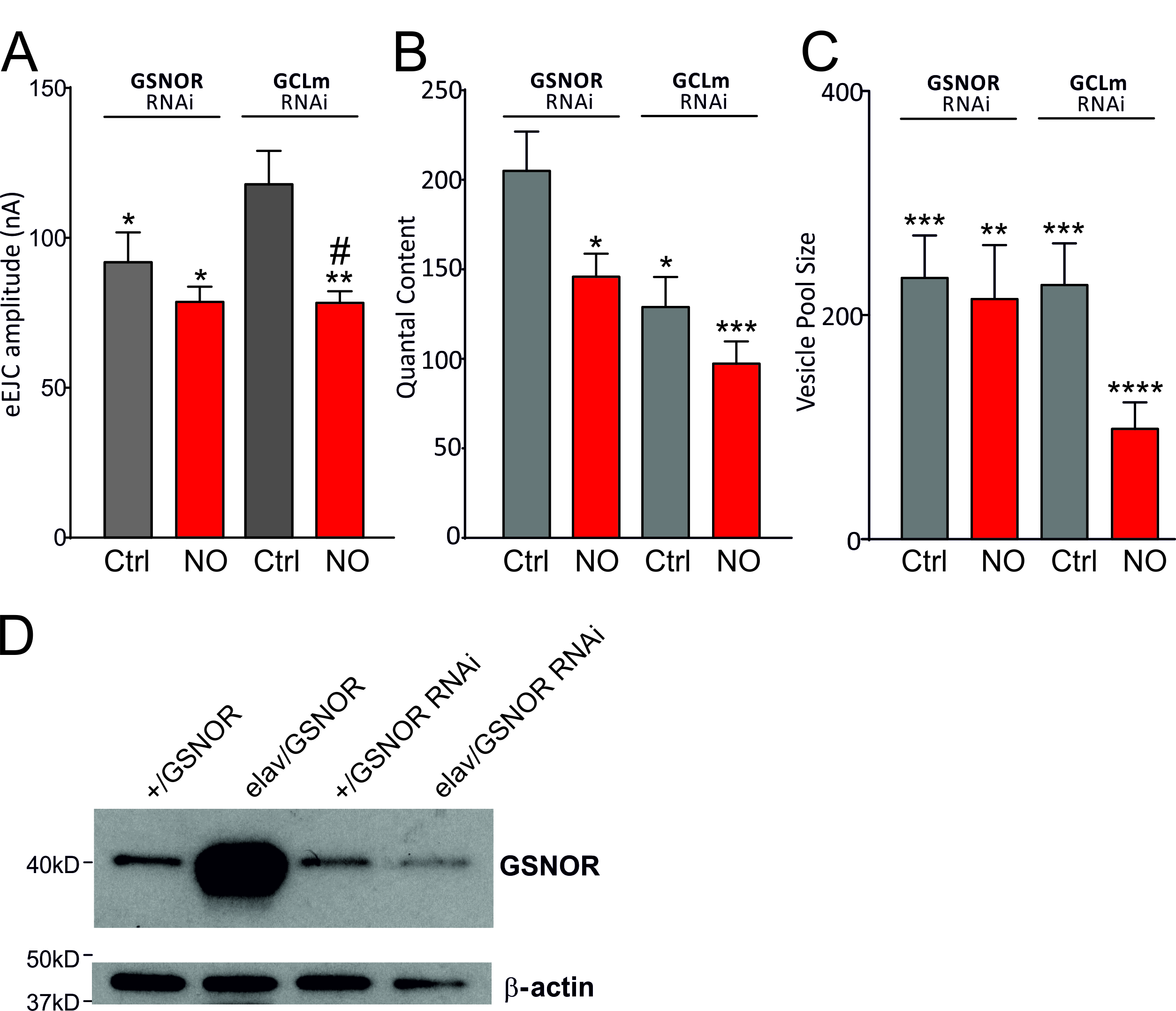

Supplement: S6 Fig — (A) Mean eEJC amplitudes (GSNOR RNAi: Ctrl: 95 ± 10 nA, NO: 85 ± 7 nA, GCLm RNAi: Ctrl: 118 ± 11 nA, NO: 78 ± 4 nA). (B) QC (GSNOR RNAi: Ctrl: 170 ± 8, NO: 139 ± 10, GCLm RNAi: Ctrl: 129 ± 17, NO: 97 ± 12) and (C) vesicle pool sizes estimated by back extrapolation from cumulative QCs (GSNOR RNAi: Ctrl: 223 ± 38, NO: 214 ± 48, GCLm RNAi: Ctrl: 228 ± 38, NO: 98 ± 24), all for genotypes indicated. (E) Western blot analysis of GSNOR (fdh31, RNAi-fdh25) expression in genotypes indicated. The raw data for this figure can be found in S9 Data. Data denote mean ± SEM, *p < 0.05, **p < 0.01, ***p < 0.001, ****p < 0.0001 versus w1118 Ctrl, #p < 0.05 versus its Ctrl, ANOVA with post hoc Tukey-Kramer was used for comparisons, n = 10–8 NMJs. Ctrl, control; eEJC, evoked EJC; EJC, excitatory junction current; fdh, formaldehyde dehydrogenase; GCLm, glutamate-cysteine ligase modifier subunit M; GSNOR, S-nitrosoglutathione reductase; NMJ, neuromuscular junction; NO, nitric oxide; QC, quantal content; RNAi, RNA interference. (TIF) [file pbio.2003611.s006.tif]

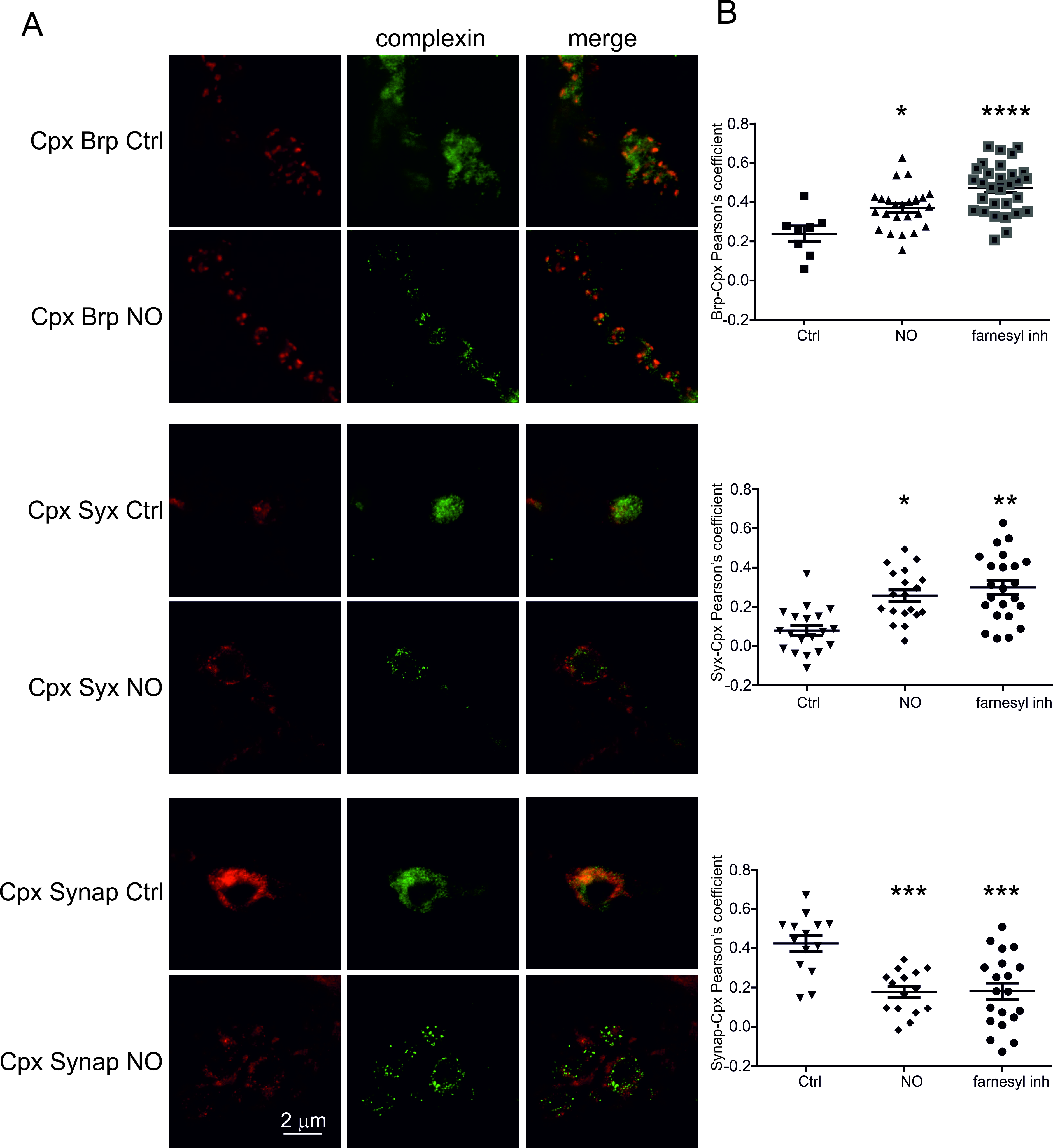

Supplement: S7 Fig — (A) Representative STED confocal images of boutons from Ctrl larvae and those exposed to NO donor. (B) Co-localization analysis reveals Pearson’s coefficients for indicated conditions (Cpx-Brp: Ctrl: 0.24 ± 0.04, NO: 0.37 ± 0.02, farnesyl inh: 0.47 ± 0.02, Cpx-Syx: Ctrl: 0.12 ± 0.04, NO: 0.26 ± 0.03, farnesyl inh: 0.30 ± 0.04, Cpx-Synap: Ctrl: 0.42 ± 0.04, NO: 0.18 ± 0.03, farnesyl inh: 0.18 ± 0.04), n = 8–30 boutons; data denote mean ± SEM, *p < 0.05, **p < 0.01, ***p < 0.001, ****p < 0.0001. ANOVA with post hoc Tukey-Kramer was used for comparisons. The raw data for this figure can be found in S9 Data. Brp, Bruchpilot; cpx, complexin; Ctrl, conrol; NO, nitric oxide; SNARE, soluble N-ethyl-maleimide-sensitive fusion protein Attachment Protein Receptor; STED, stimulated emission depletion; Synap, synaptotagmin; Syx, syntaxin. (TIF) [file pbio.2003611.s007.tif]

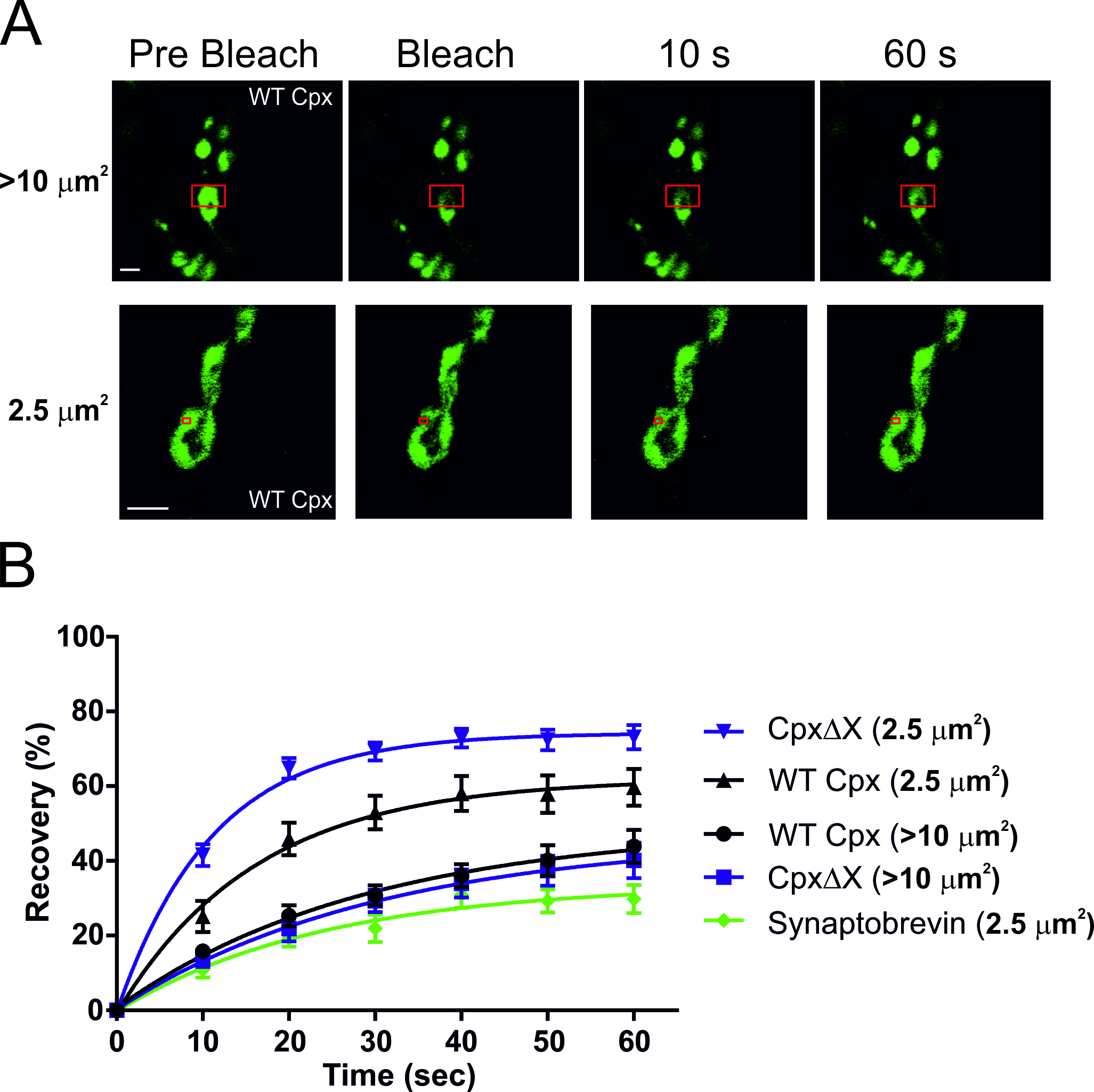

Supplement: S8 Fig — (A) Representative images of boutons expressing WT cpx-GFP before bleaching and at different time points after photo bleaching. Top row shows recordings with a bleaching area roughly the size of half a bouton (>10 μm2); bottom row shows images using a bleaching area of 2.5 μm2. (B) Analysis of recovery from bleach shows faster time constants using the smaller bleaching area compared to half-bouton bleach. Using the smaller bleaching areas, there is a pronounced difference between WT and mutant cpx. Synaptobrevin was used as a control for photo bleaching associated with vesicular movement. Note, images and analysis for the 2.5 μm2 bleaching areas are the same as in Fig 7C; scale bar: 2 μm. The raw data for this figure can be found in S9 Data. cpx, complexin; FRAP, fluorescence recovery after photobleaching; GFP, green fluorescent protein; WT, wild-type. (TIF) [file pbio.2003611.s008.tif]

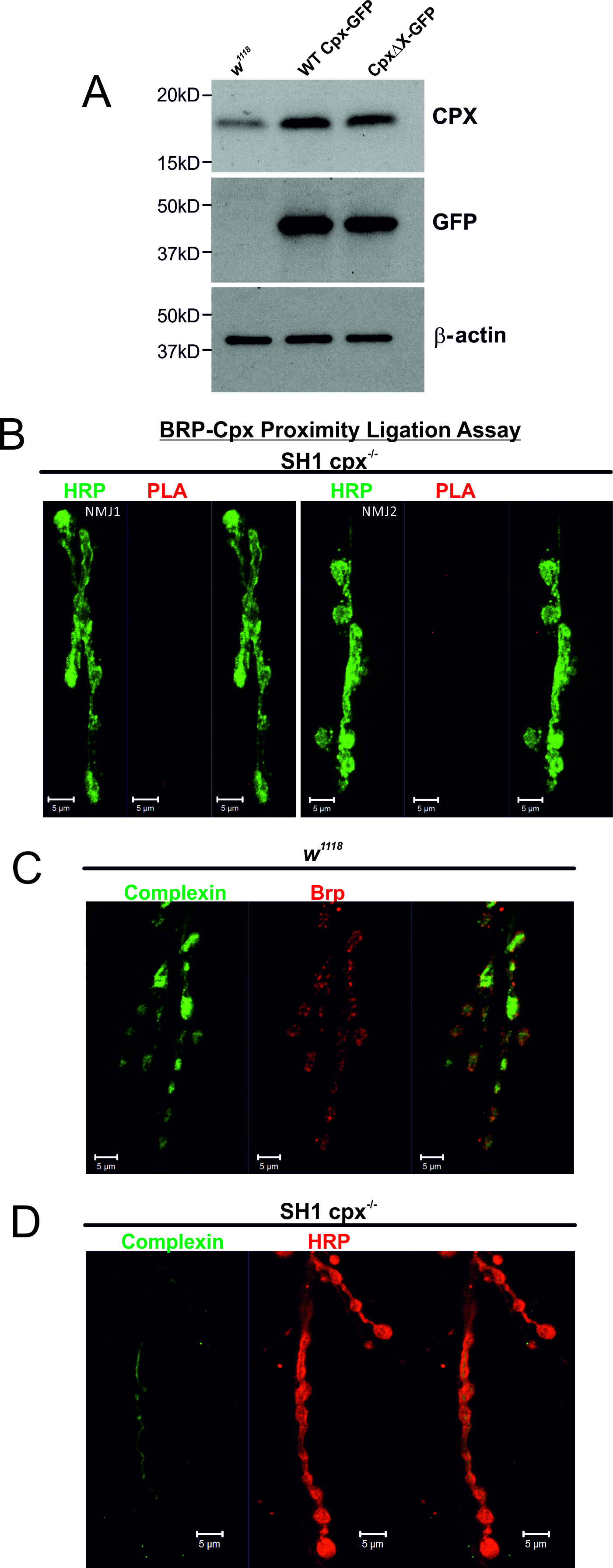

Supplement: S9 Fig — (A) Western blot analysis shows expression levels of cpx, GFP, and β-actin in w1118, WT cpx-GFP and cpxΔX-GFP lines. (B) Representative images of Brp-Cpx PLA in 2 example cpxSH1 NMJs show no PLA signal. (C) Confocal single plane images of a w1118 NMJ stained for cpx and Brp and (D) confocal single plane image of a cpxSH1 larva that does not express cpx. Brp, Bruchpilot; cpx, complexin; GFP, green fluorescent protein; NMJ, neuromuscular junction; PLA, proximity ligation assay; WT, wild-type. (TIF) [file pbio.2003611.s009.tif]

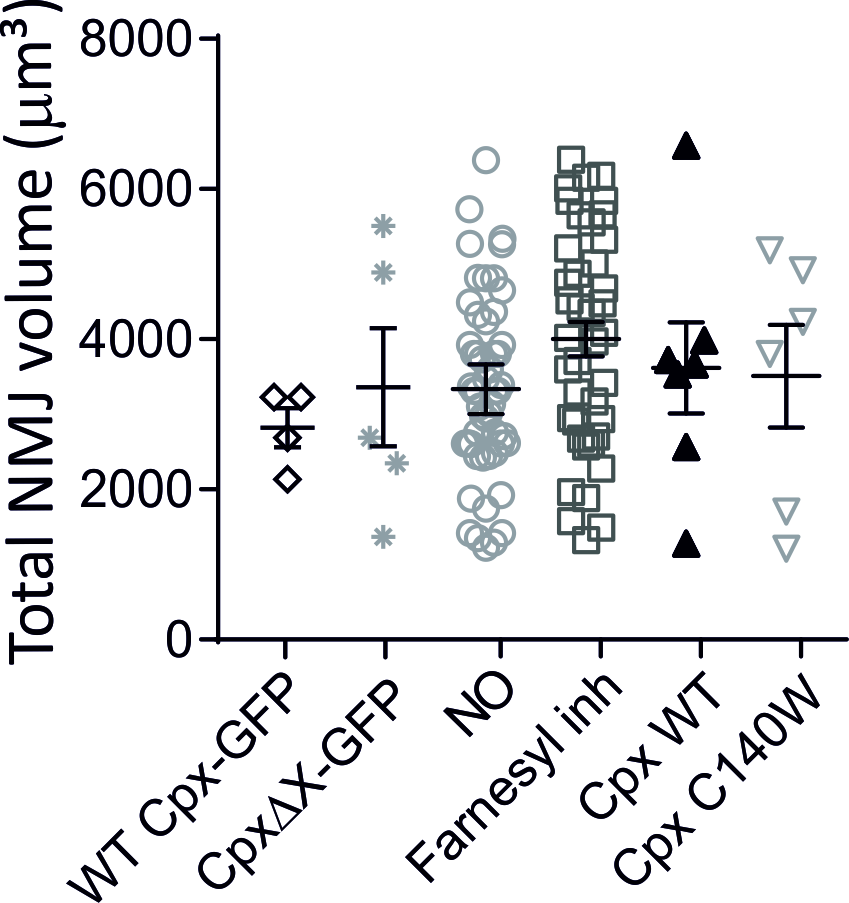

Supplement: S10 Fig — NMJs for different genotypes and conditions have similar volumes compared to Ctrls (WT Cpx-GFP [Cpx2A]: 2,857 ± 261 μm3, CpxΔX-GFP [Cpx1257]: 3,395 ± 787 μm3, NO treatment: 3,400 ± 228 μm3, farnesyl inh: 3,973 ± 306 μm3, Cpx WT: 3,617 ± 875 μm3, CpxC140W: 3,508 ± 707 μm3). Data denote mean ± SEM; ANOVA with post hoc Tukey-Kramer was used for comparisons versus w1118 WT, p > 0.05. The raw data for this figure can be found in S9 Data. Cpx, complexin; Ctrl, control; GFP, green fluorescent protein; NMJ, neuromuscular junction; NO, nitric oxide; WT, wild-type. (TIF) [file pbio.2003611.s010.tif]
